# Supplementary material for: Ruscus aculeatus extract promotes RNase 7 expression through ERK activation following inhibition of late-phase autophagy in primary human keratinocytes
Source: PLoS One. 2024 Dec 3;19(12):e0314873. doi: 10.1371/journal.pone.0314873 (PMC11614269; doi:10.1371/journal.pone.0314873)
Supplement: S1 Fig — (PPTX) [file pone.0314873.s002.pptx]

## Slide 1
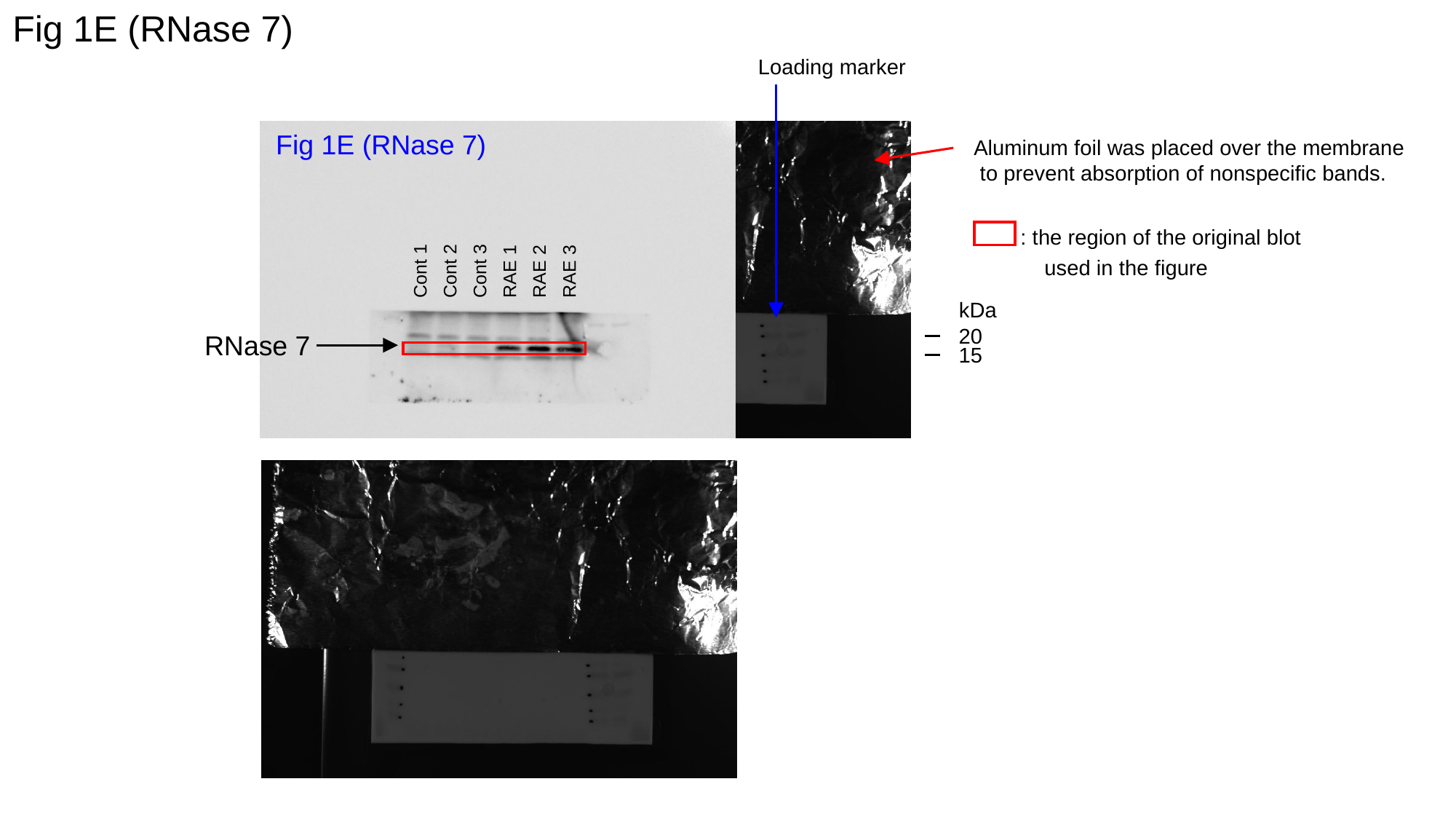

Fig 1E (RNase 7)
Loading marker
Fig 1E (RNase 7)
Aluminum foil was placed over the membrane
 to prevent absorption of nonspecific bands.
: the region of the original blot
 used in the figure
Cont 1
Cont 2
Cont 3
RAE 1
RAE 2
RAE 3
kDa
20
15
RNase 7

## Slide 2
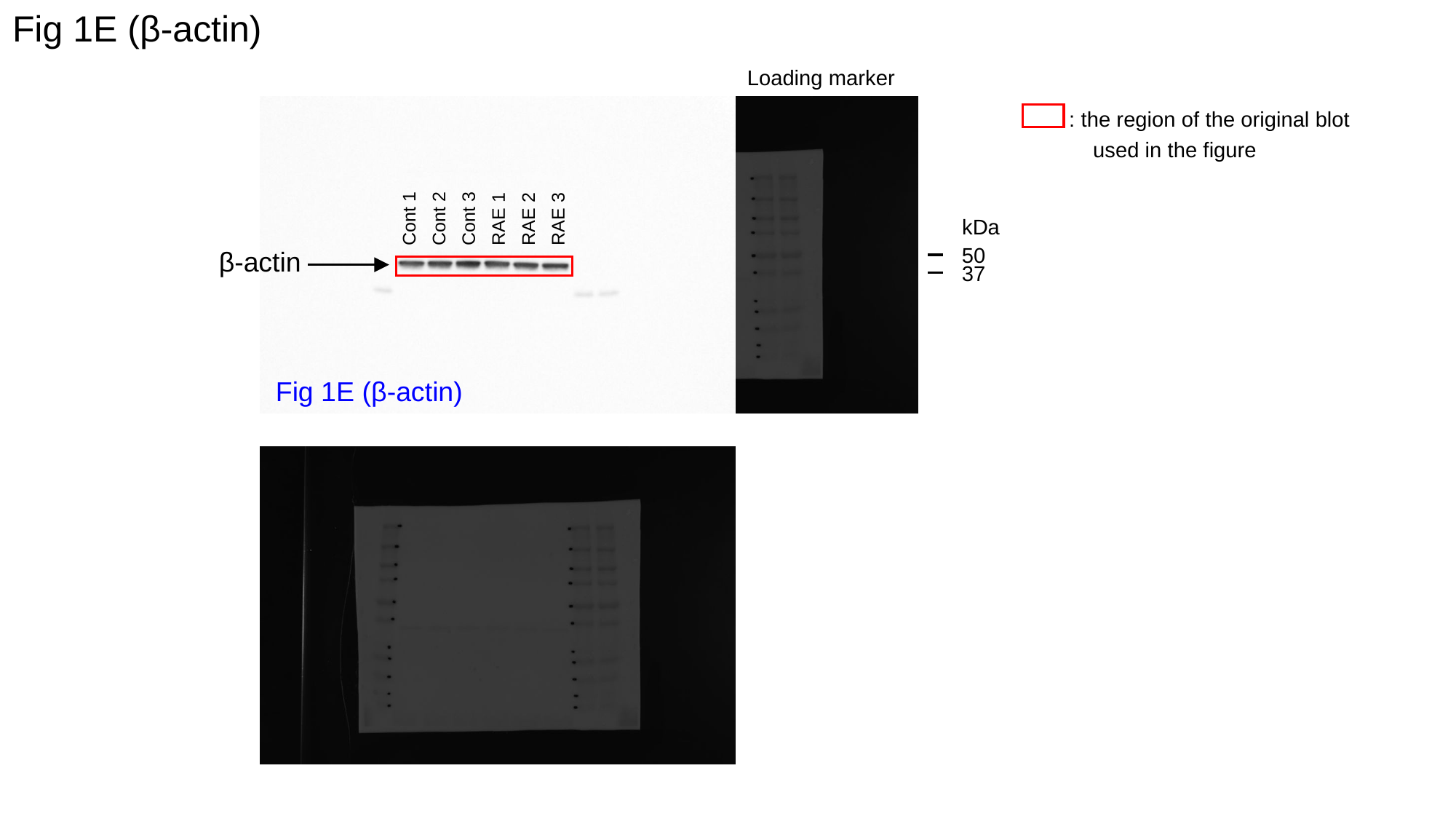

Fig 1E (β-actin)
Loading marker
: the region of the original blot
 used in the figure
Cont 1
Cont 2
Cont 3
RAE 1
RAE 2
RAE 3
kDa
50
37
β-actin
Fig 1E (β-actin)

## Slide 3
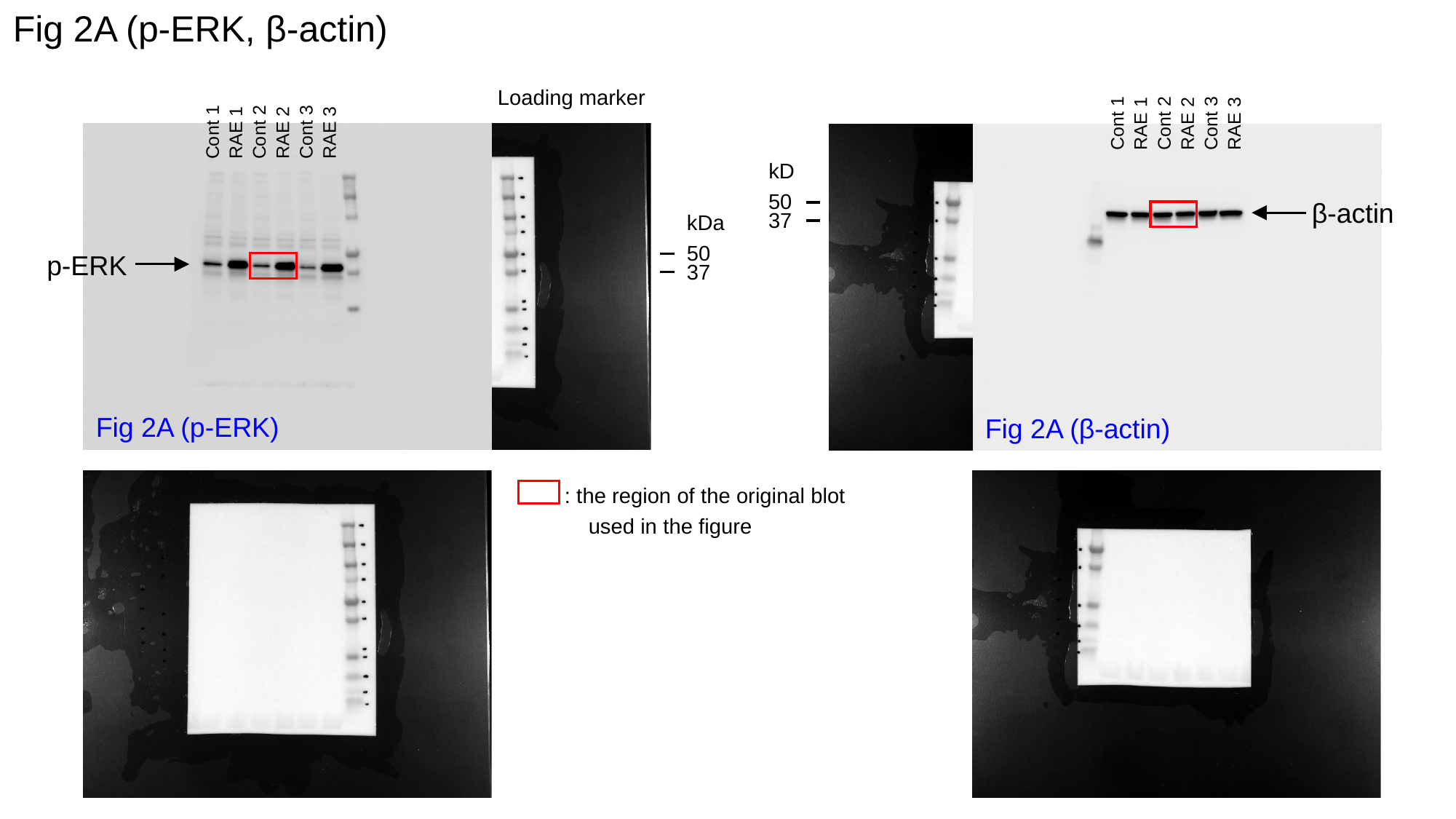

Fig 2A (p-ERK, β-actin)
Loading marker
Cont 3
Cont 1
Cont 2
RAE 1
RAE 2
RAE 3
kDa
50
37
p-ERK
Fig 2A (p-ERK)
Cont 3
Cont 1
Cont 2
RAE 1
RAE 2
RAE 3
kD
50
37
β-actin
Fig 2A (β-actin)
: the region of the original blot
 used in the figure

## Slide 4
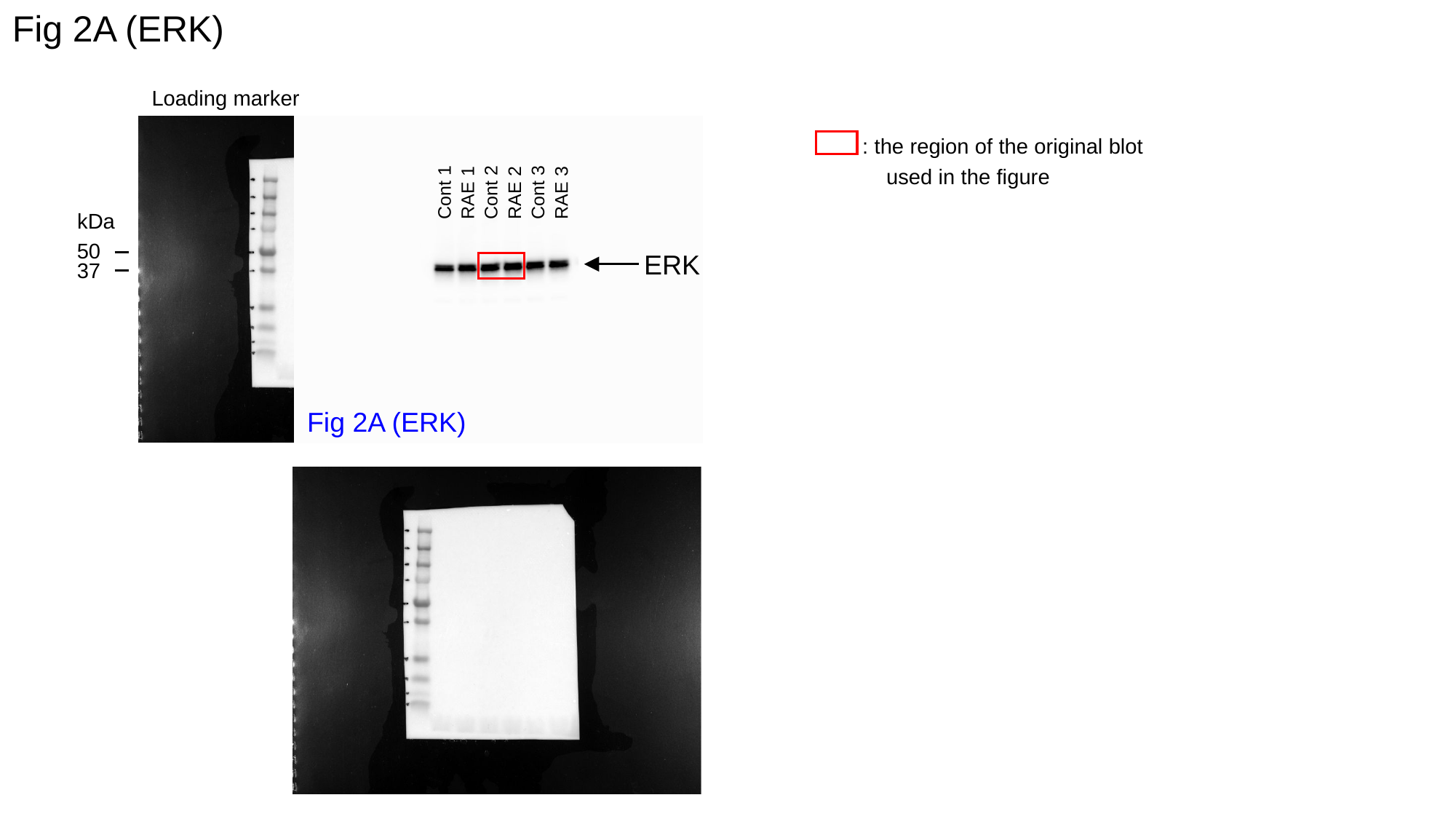

Fig 2A (ERK)
Loading marker
Cont 3
Cont 1
Cont 2
RAE 1
RAE 2
RAE 3
kDa
50
37
ERK
Fig 2A (ERK)
: the region of the original blot
 used in the figure

## Slide 5
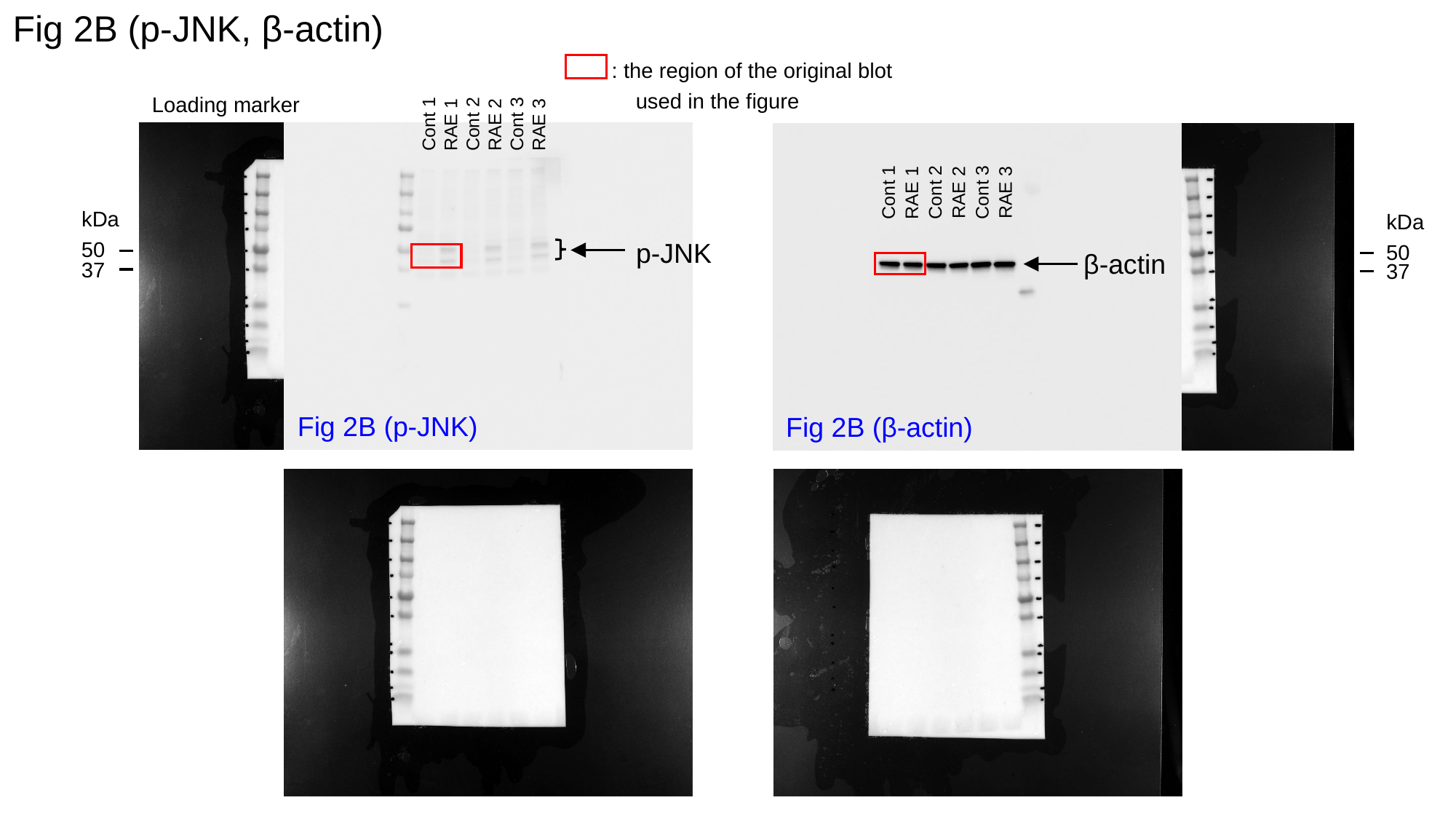

Fig 2B (p-JNK, β-actin)
: the region of the original blot
 used in the figure
Loading marker
Cont 3
Cont 1
Cont 2
RAE 1
RAE 2
RAE 3
kDa
50
37
p-JNK
Fig 2B (p-JNK)
Cont 3
Cont 1
Cont 2
RAE 1
RAE 2
RAE 3
kDa
50
37
β-actin
Fig 2B (β-actin)

## Slide 6
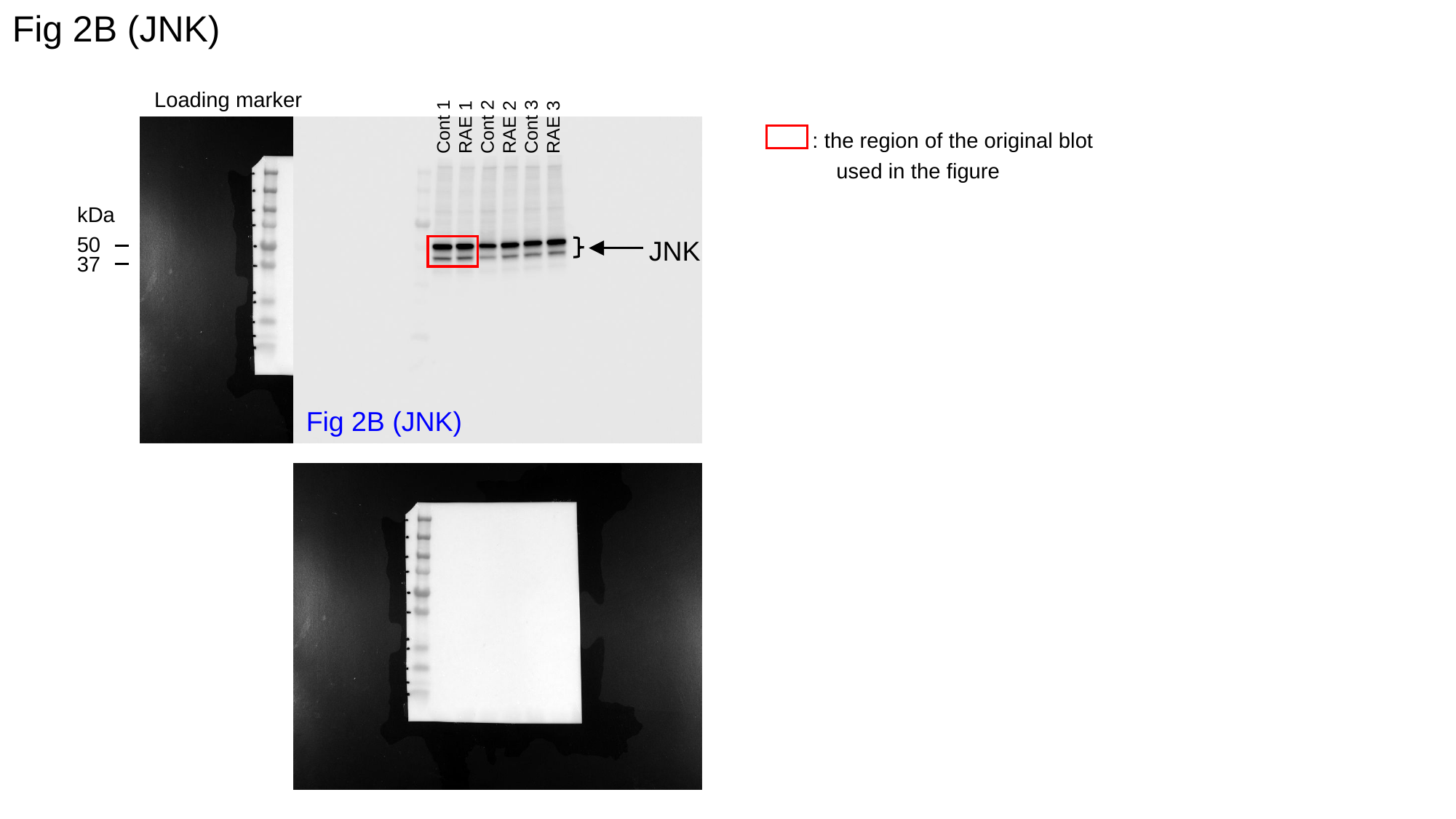

Fig 2B (JNK)
Loading marker
Cont 3
Cont 1
Cont 2
RAE 1
RAE 2
RAE 3
kDa
50
37
JNK
Fig 2B (JNK)
: the region of the original blot
 used in the figure

## Slide 7
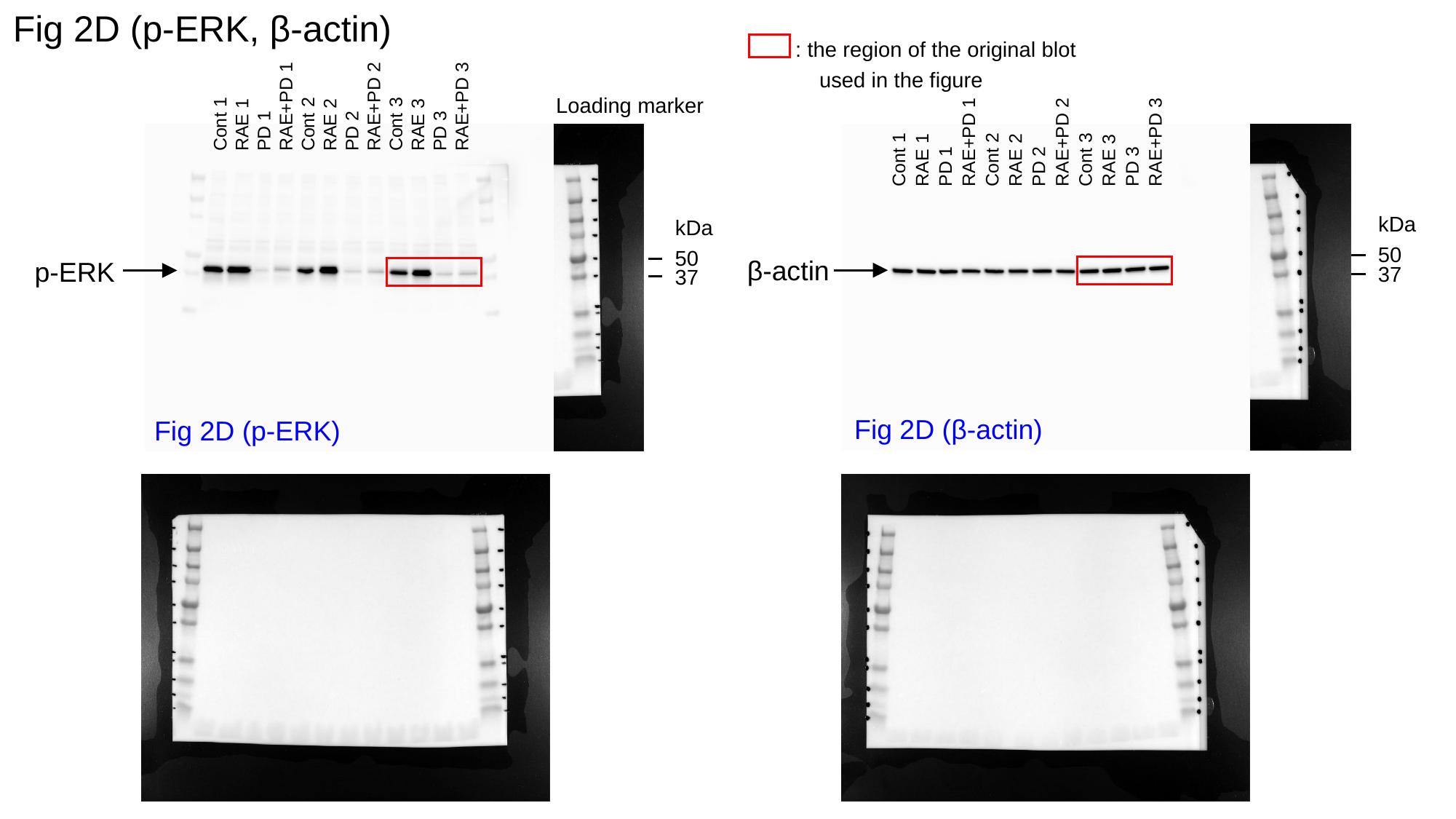

Fig 2D (p-ERK, β-actin)
: the region of the original blot
 used in the figure
RAE+PD 1
RAE+PD 2
RAE+PD 3
Cont 1
Cont 2
Cont 3
RAE 1
RAE 2
RAE 3
PD 1
PD 2
PD 3
Loading marker
kDa
50
37
p-ERK
Fig 2D (p-ERK)
RAE+PD 1
RAE+PD 2
RAE+PD 3
Cont 1
Cont 2
Cont 3
RAE 1
RAE 2
RAE 3
PD 1
PD 2
PD 3
kDa
50
37
β-actin
Fig 2D (β-actin)

## Slide 8
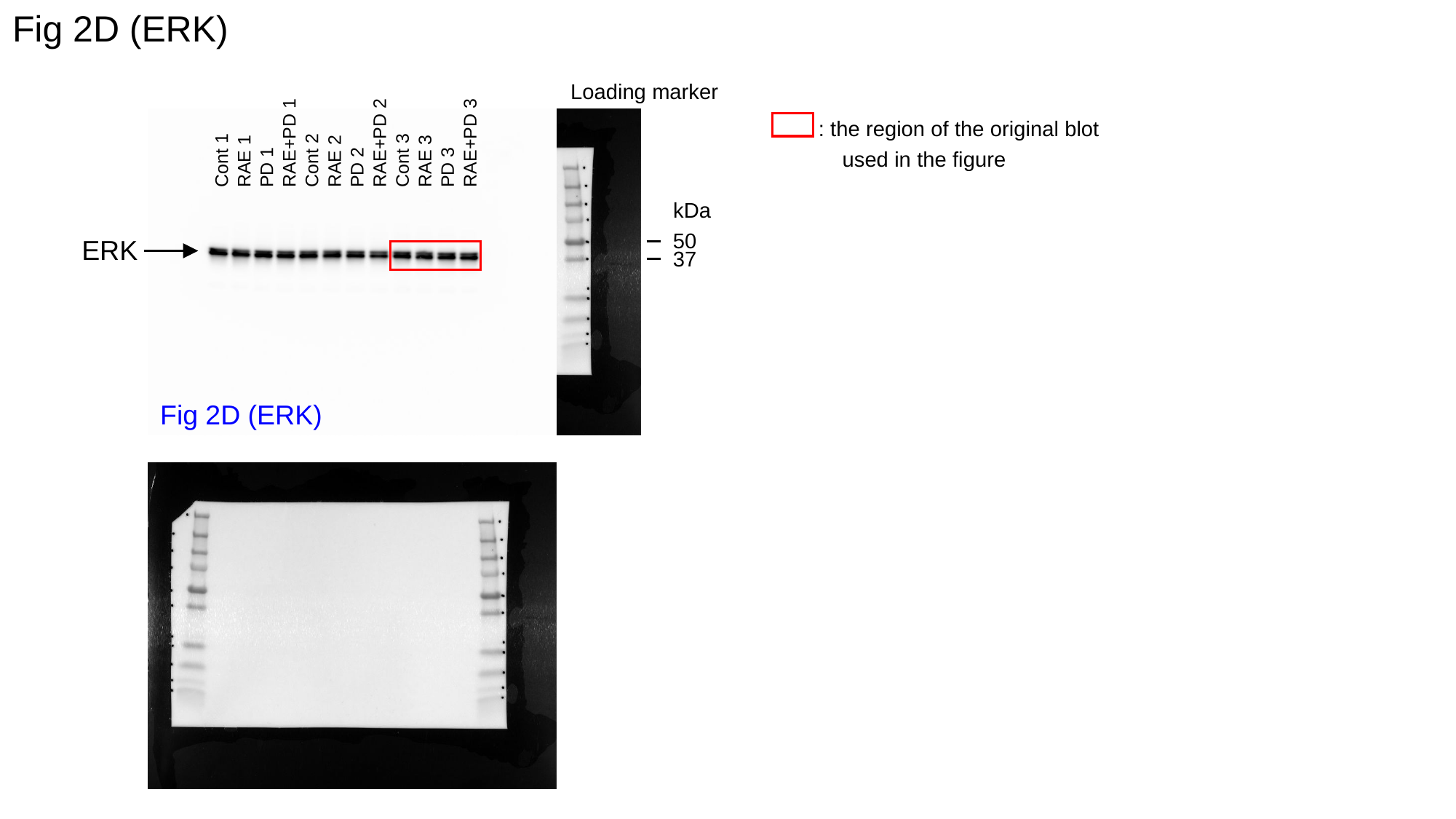

Fig 2D (ERK)
Loading marker
RAE+PD 1
RAE+PD 2
RAE+PD 3
Cont 1
Cont 2
Cont 3
RAE 1
RAE 2
RAE 3
PD 1
PD 2
PD 3
kDa
50
37
ERK
Fig 2D (ERK)
: the region of the original blot
 used in the figure

## Slide 9
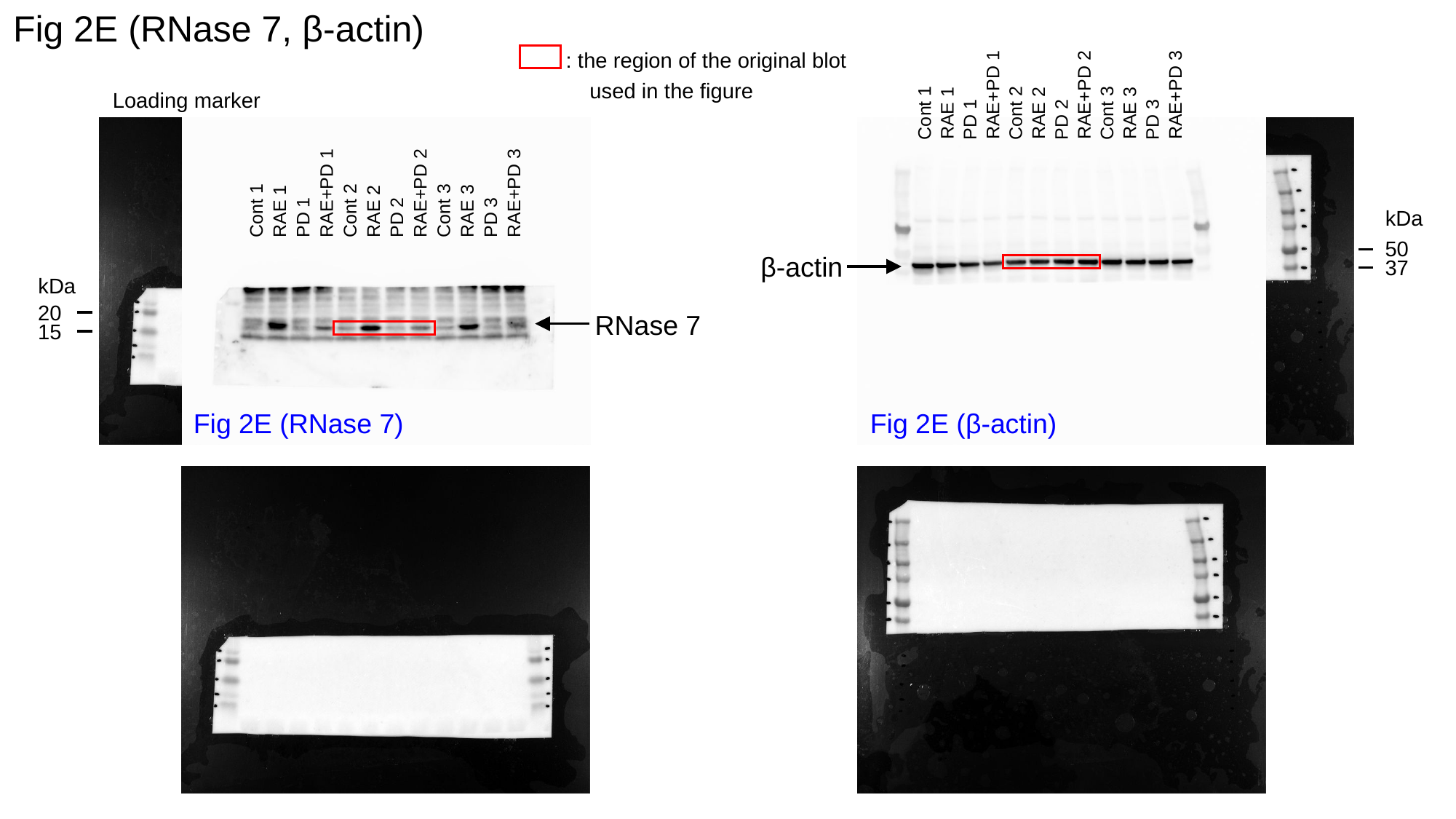

Fig 2E (RNase 7, β-actin)
: the region of the original blot
 used in the figure
RAE+PD 1
RAE+PD 2
RAE+PD 3
Cont 1
Cont 2
Cont 3
RAE 1
RAE 2
RAE 3
PD 1
PD 2
PD 3
kDa
50
37
β-actin
Fig 2E (β-actin)
Loading marker
RAE+PD 1
RAE+PD 2
RAE+PD 3
Cont 1
Cont 2
Cont 3
RAE 1
RAE 2
RAE 3
PD 1
PD 2
PD 3
kDa
20
15
RNase 7
Fig 2E (RNase 7)

## Slide 10
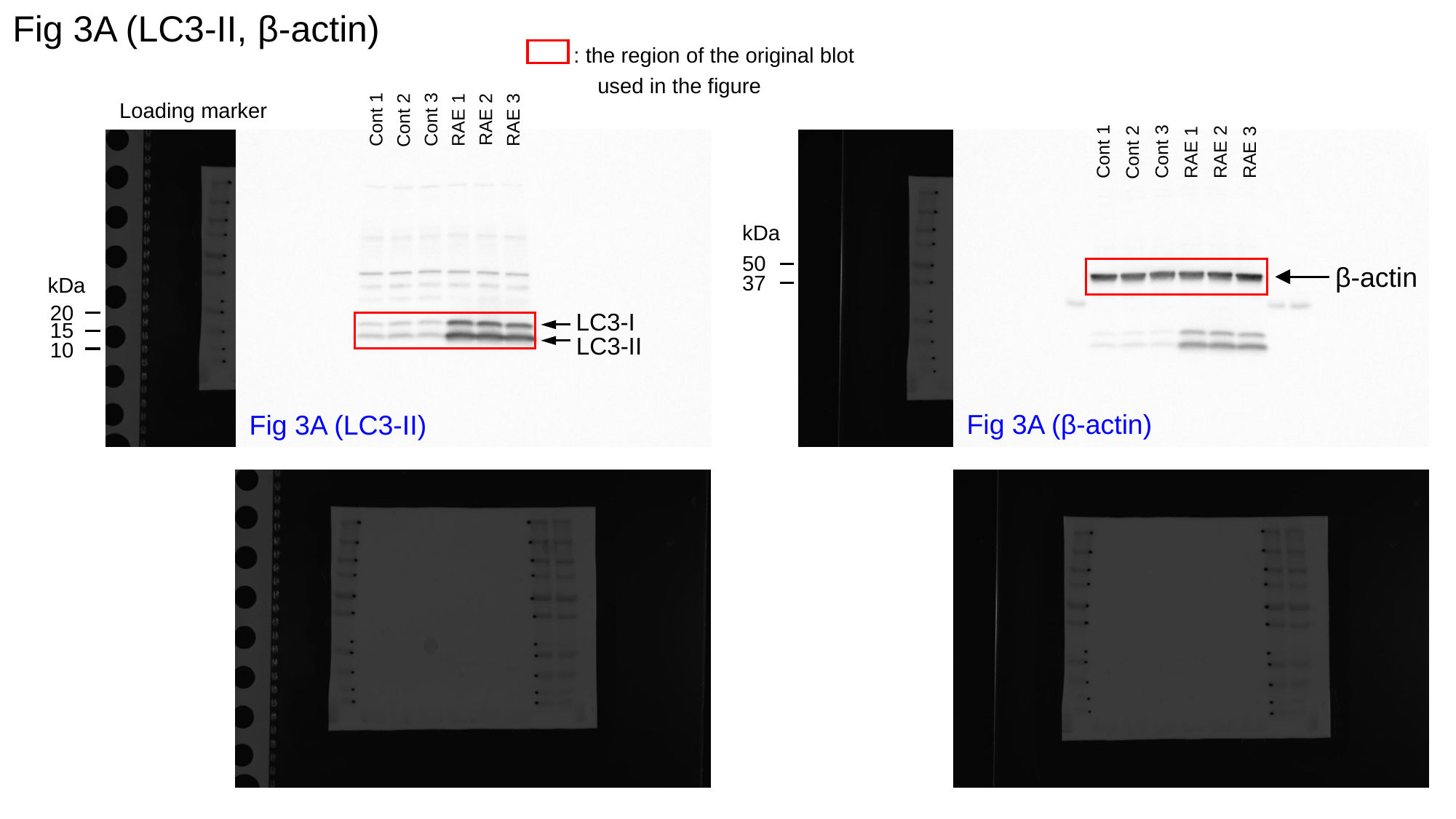

Fig 3A (LC3-II, β-actin)
: the region of the original blot
 used in the figure
RAE 2
Cont 1
Cont 3
Cont 2
RAE 1
RAE 3
Loading marker
kDa
20
15
10
LC3-I
LC3-II
Fig 3A (LC3-II)
RAE 2
Cont 1
Cont 3
Cont 2
RAE 1
RAE 3
kDa
50
37
β-actin
Fig 3A (β-actin)

## Slide 11
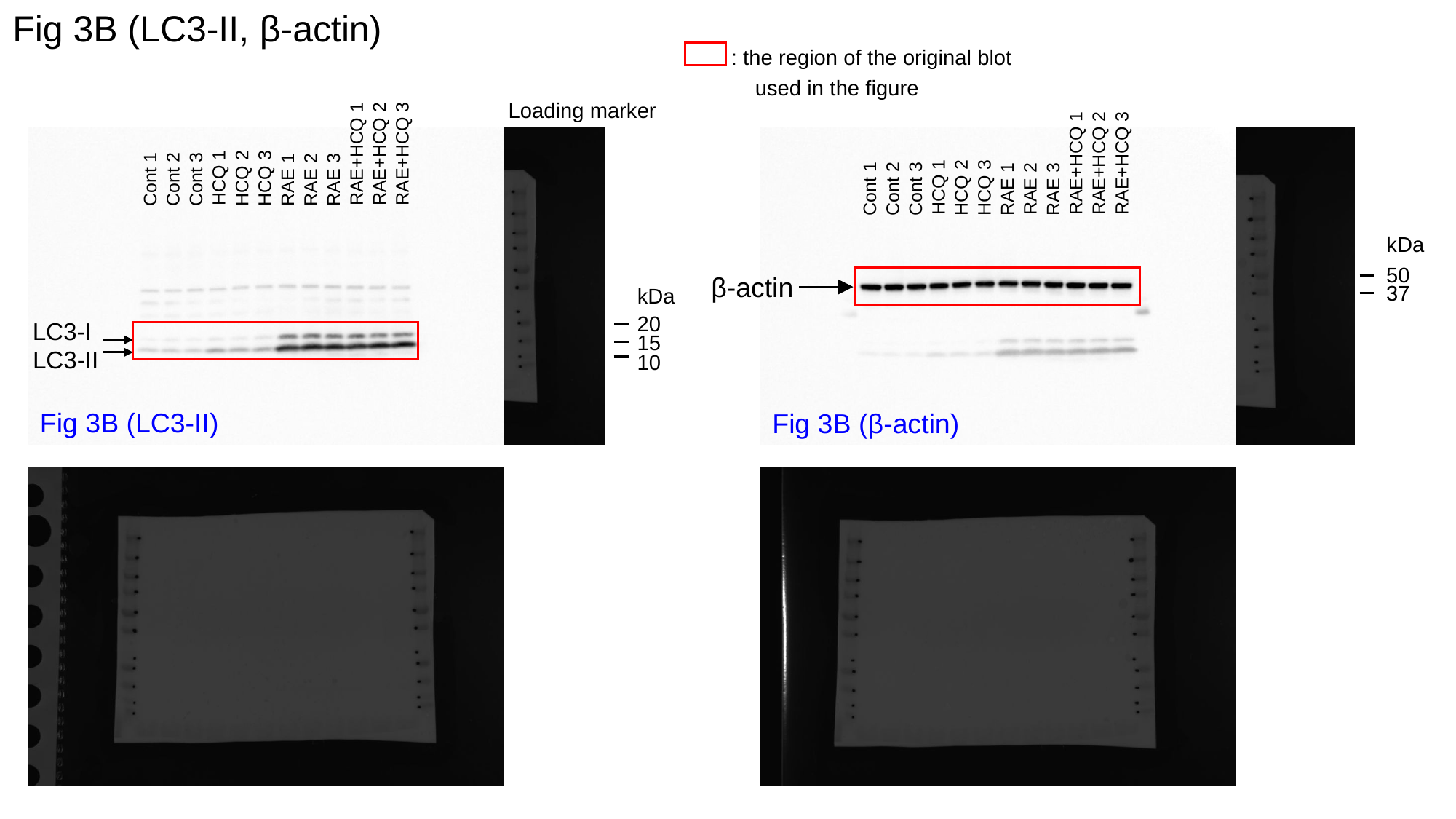

Fig 3B (LC3-II, β-actin)
: the region of the original blot
 used in the figure
RAE+HCQ 1
RAE+HCQ 2
RAE+HCQ 3
HCQ 1
HCQ 2
HCQ 3
Cont 1
Cont 2
Cont 3
RAE 1
RAE 2
RAE 3
Loading marker
kDa
20
15
10
LC3-I
LC3-II
Fig 3B (LC3-II)
RAE+HCQ 1
RAE+HCQ 2
RAE+HCQ 3
HCQ 1
HCQ 2
HCQ 3
Cont 1
Cont 2
Cont 3
RAE 1
RAE 2
RAE 3
kDa
50
37
β-actin
Fig 3B (β-actin)

## Slide 12
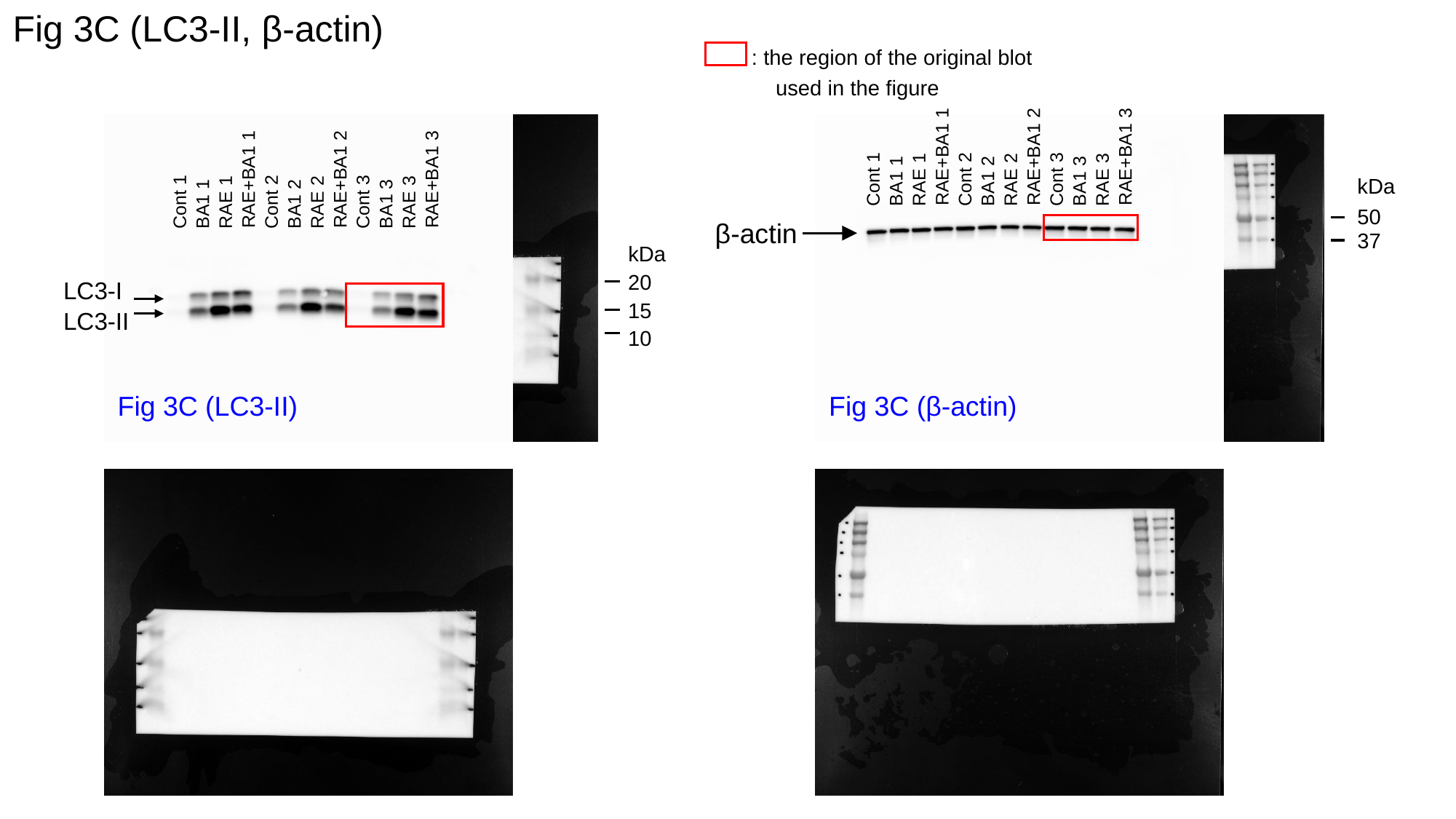

Fig 3C (LC3-II, β-actin)
: the region of the original blot
 used in the figure
RAE+BA1 2
RAE+BA1 1
RAE+BA1 3
Cont 1
Cont 2
Cont 3
RAE 1
RAE 2
RAE 3
BA1 1
BA1 2
BA1 3
kDa
50
37
β-actin
Fig 3C (β-actin)
RAE+BA1 2
RAE+BA1 1
RAE+BA1 3
Cont 1
Cont 2
Cont 3
RAE 1
RAE 2
RAE 3
BA1 1
BA1 2
BA1 3
kDa
20
15
10
LC3-I
LC3-II
Fig 3C (LC3-II)

## Slide 13
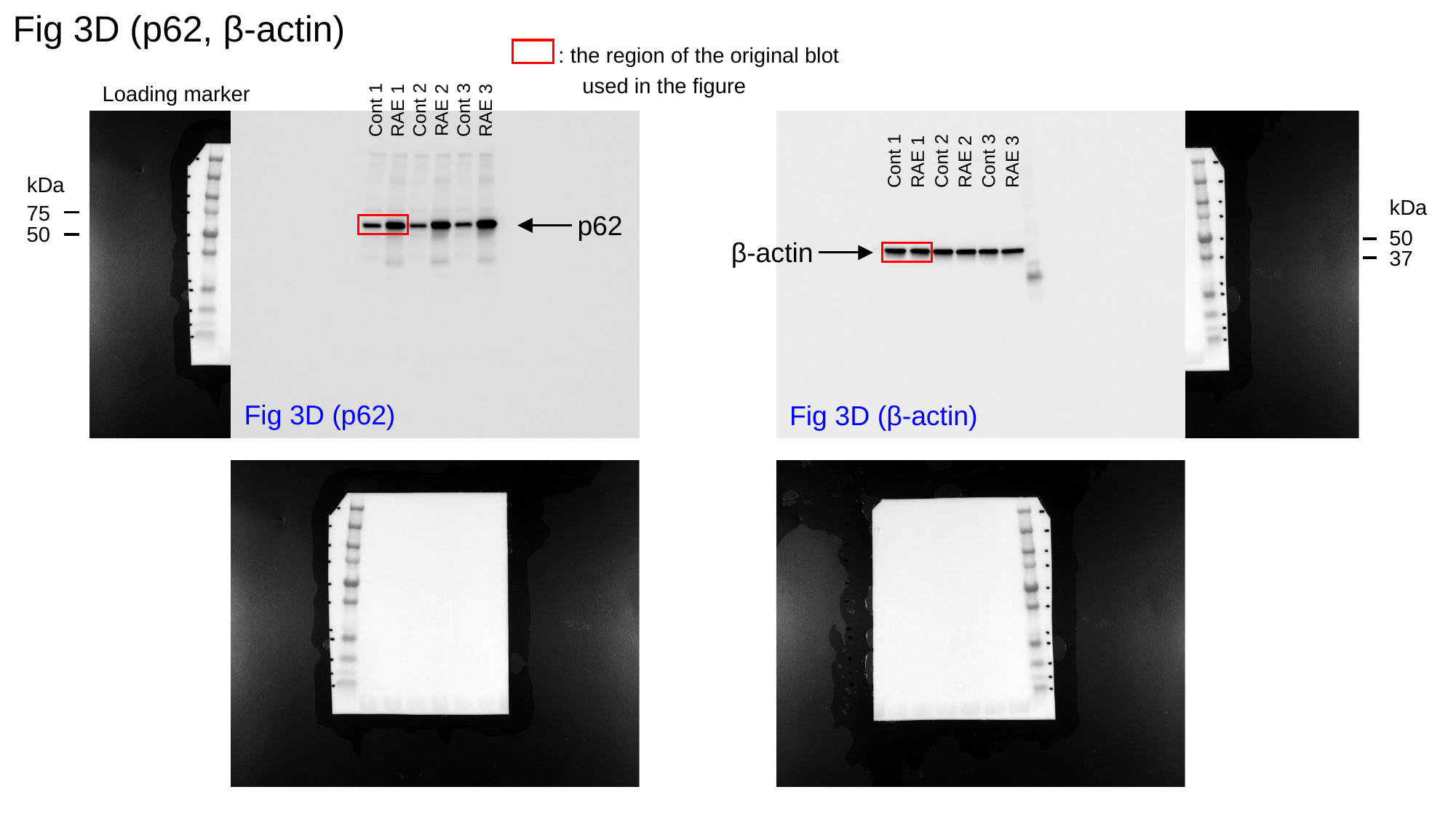

Fig 3D (p62, β-actin)
: the region of the original blot
 used in the figure
Cont 3
Cont 1
Cont 2
RAE 1
RAE 2
RAE 3
Loading marker
kDa
75
50
p62
Fig 3D (p62)
Cont 3
Cont 1
Cont 2
RAE 1
RAE 2
RAE 3
kDa
50
37
β-actin
Fig 3D (β-actin)

## Slide 14
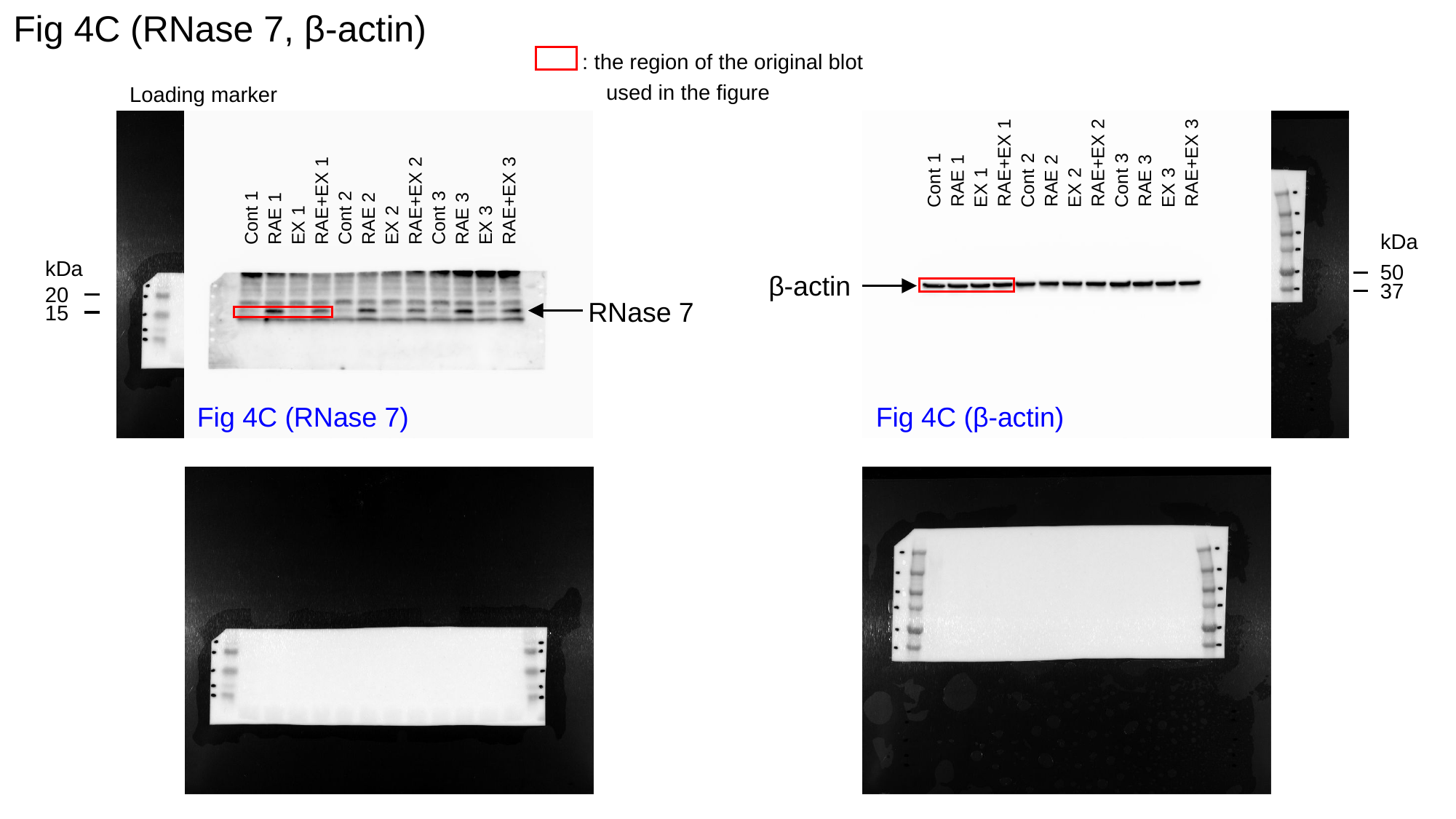

Fig 4C (RNase 7, β-actin)
: the region of the original blot
 used in the figure
Loading marker
RAE+EX 3
RAE+EX 1
RAE+EX 2
Cont 1
Cont 2
Cont 3
RAE 1
RAE 2
RAE 3
EX 3
EX 1
EX 2
kDa
20
15
RNase 7
Fig 4C (RNase 7)
RAE+EX 1
RAE+EX 2
RAE+EX 3
Cont 1
Cont 2
Cont 3
RAE 1
RAE 2
RAE 3
EX 1
EX 2
EX 3
kDa
50
37
β-actin
Fig 4C (β-actin)

## Slide 15
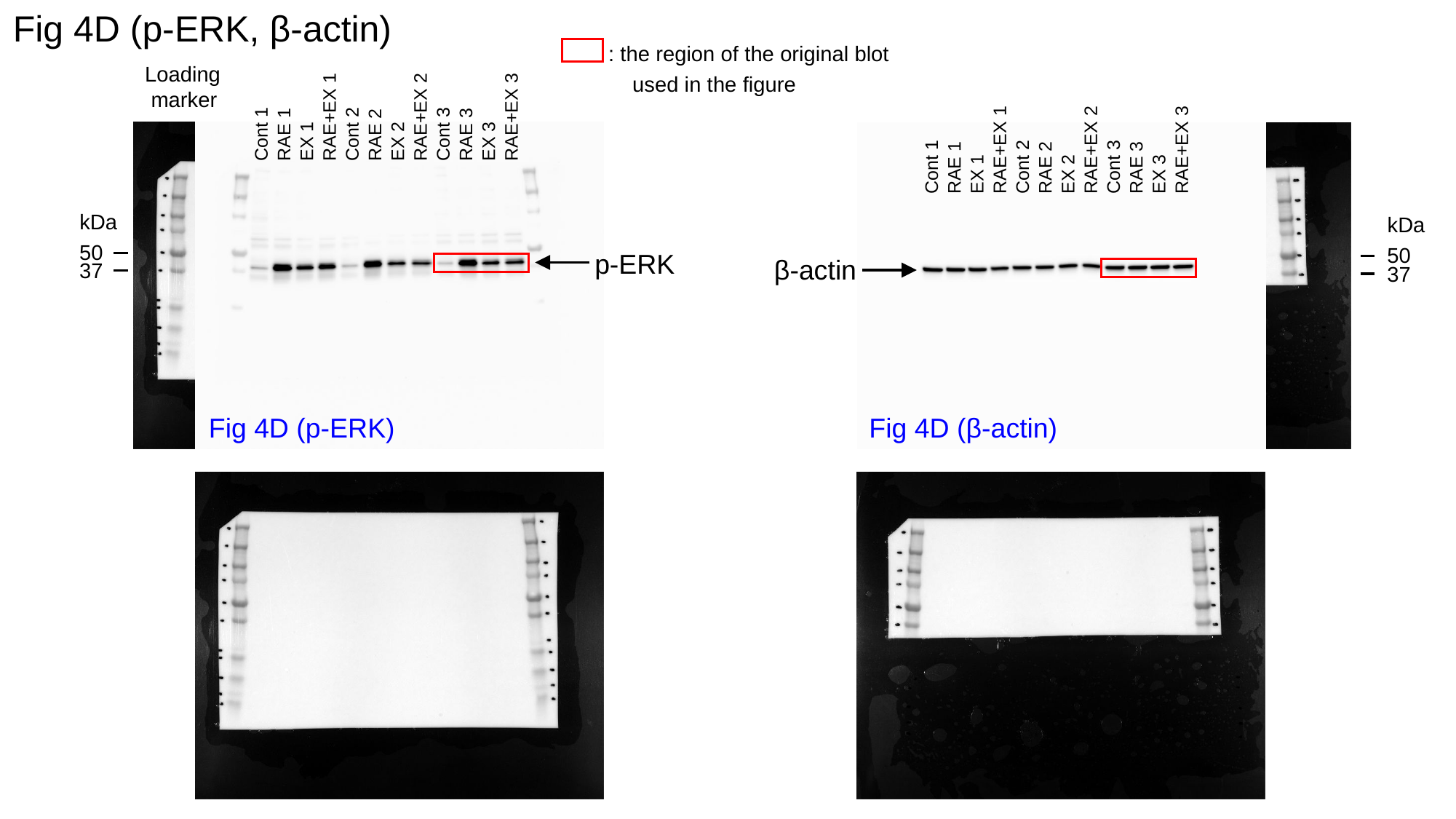

Fig 4D (p-ERK, β-actin)
: the region of the original blot
 used in the figure
Loading
 marker
RAE+EX 1
RAE+EX 2
RAE+EX 3
Cont 1
Cont 2
Cont 3
RAE 1
RAE 2
RAE 3
EX 1
EX 2
EX 3
kDa
50
37
p-ERK
Fig 4D (p-ERK)
RAE+EX 1
RAE+EX 2
RAE+EX 3
Cont 1
Cont 2
Cont 3
RAE 1
RAE 2
RAE 3
EX 1
EX 2
EX 3
kDa
50
37
β-actin
Fig 4D (β-actin)

## Slide 16
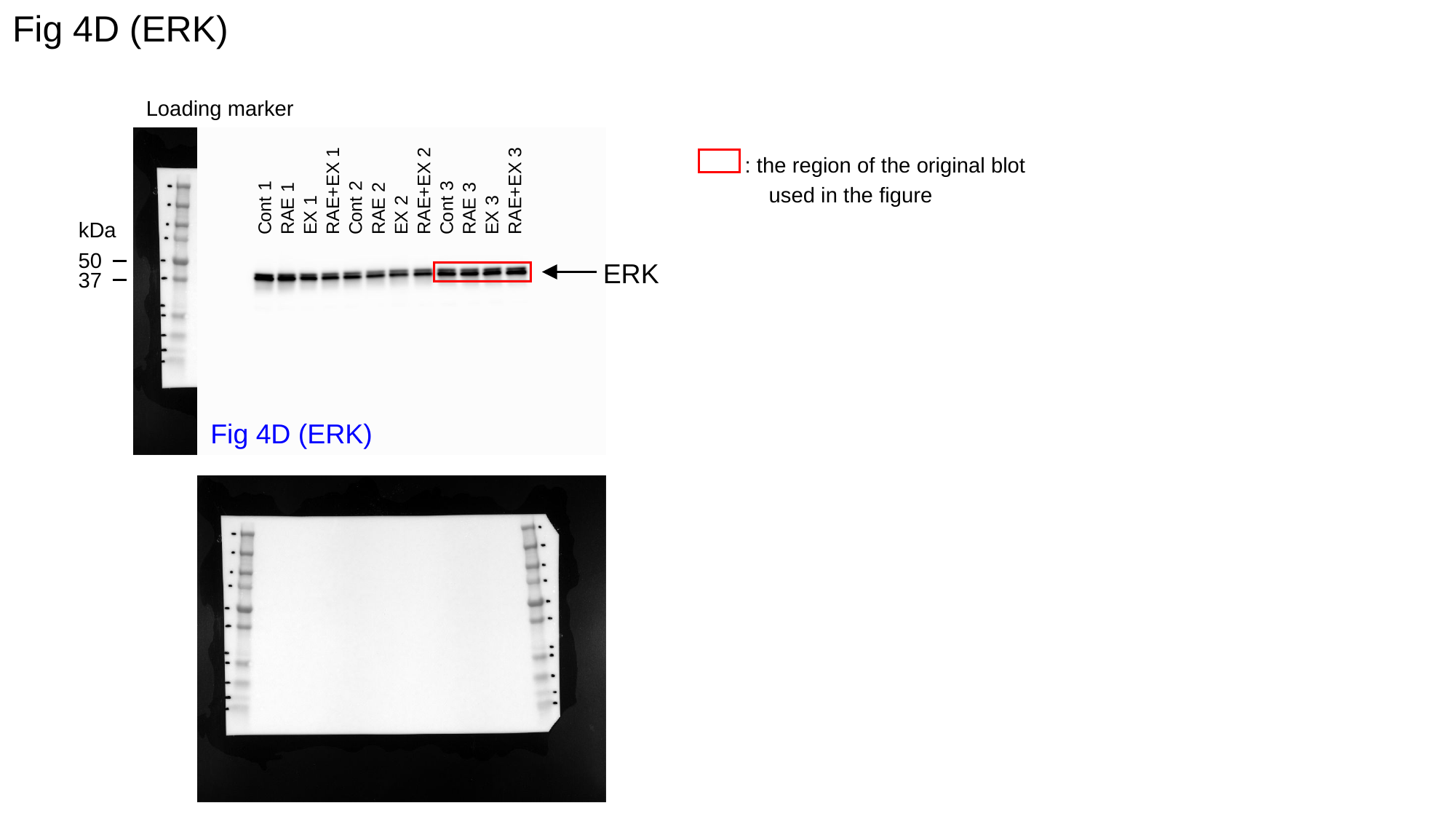

Fig 4D (ERK)
Loading marker
RAE+EX 1
RAE+EX 2
RAE+EX 3
Cont 1
Cont 2
Cont 3
RAE 1
RAE 2
RAE 3
EX 1
EX 2
EX 3
kDa
50
37
ERK
Fig 4D (ERK)
: the region of the original blot
 used in the figure

## Slide 17
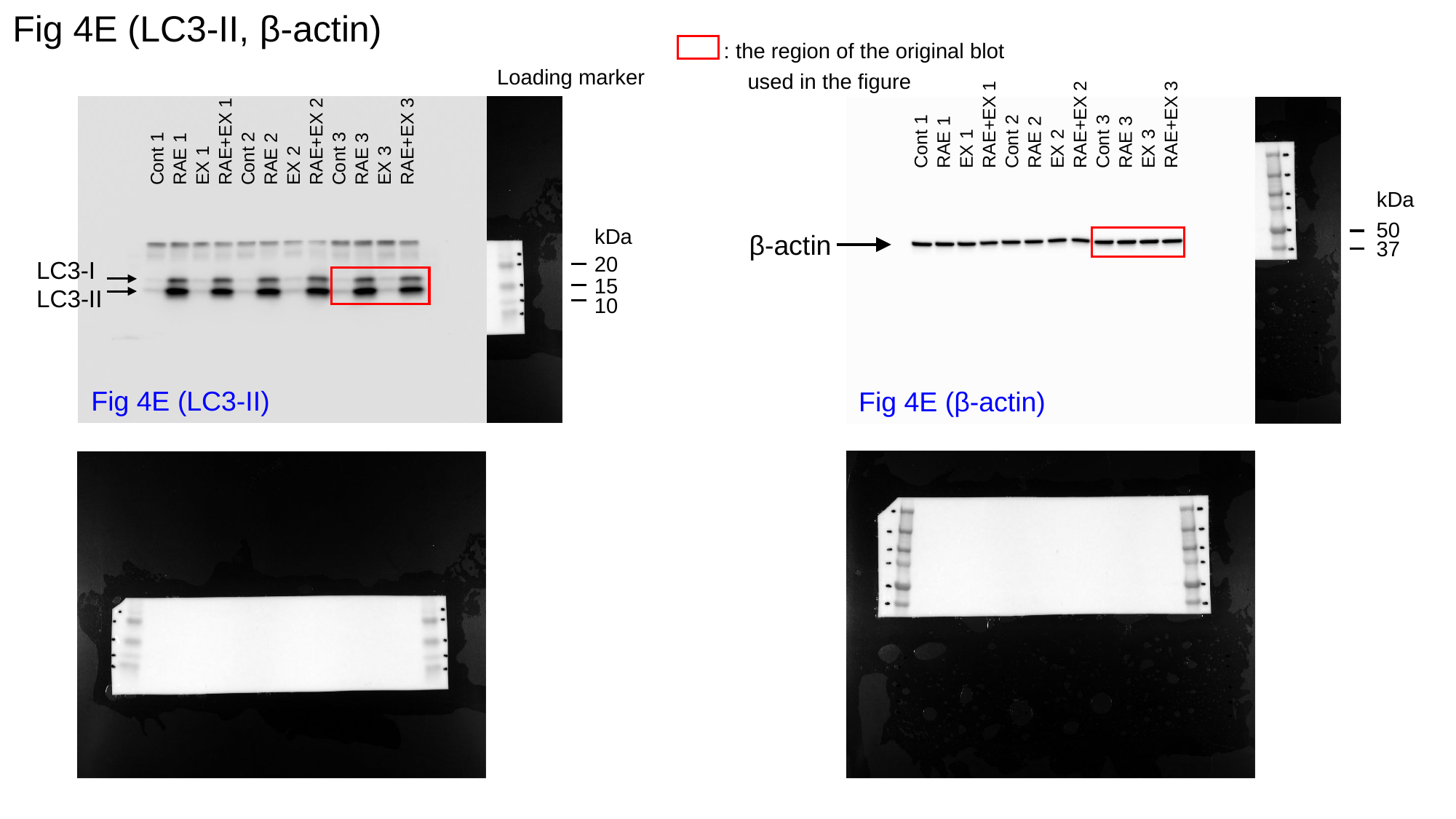

Fig 4E (LC3-II, β-actin)
: the region of the original blot
 used in the figure
Loading marker
RAE+EX 1
RAE+EX 2
RAE+EX 3
Cont 1
Cont 2
Cont 3
RAE 1
RAE 2
RAE 3
EX 1
EX 2
EX 3
kDa
20
15
10
LC3-I
LC3-II
Fig 4E (LC3-II)
RAE+EX 1
RAE+EX 2
RAE+EX 3
Cont 1
Cont 2
Cont 3
RAE 1
RAE 2
RAE 3
EX 1
EX 2
EX 3
kDa
50
37
β-actin
Fig 4E (β-actin)

## Slide 18
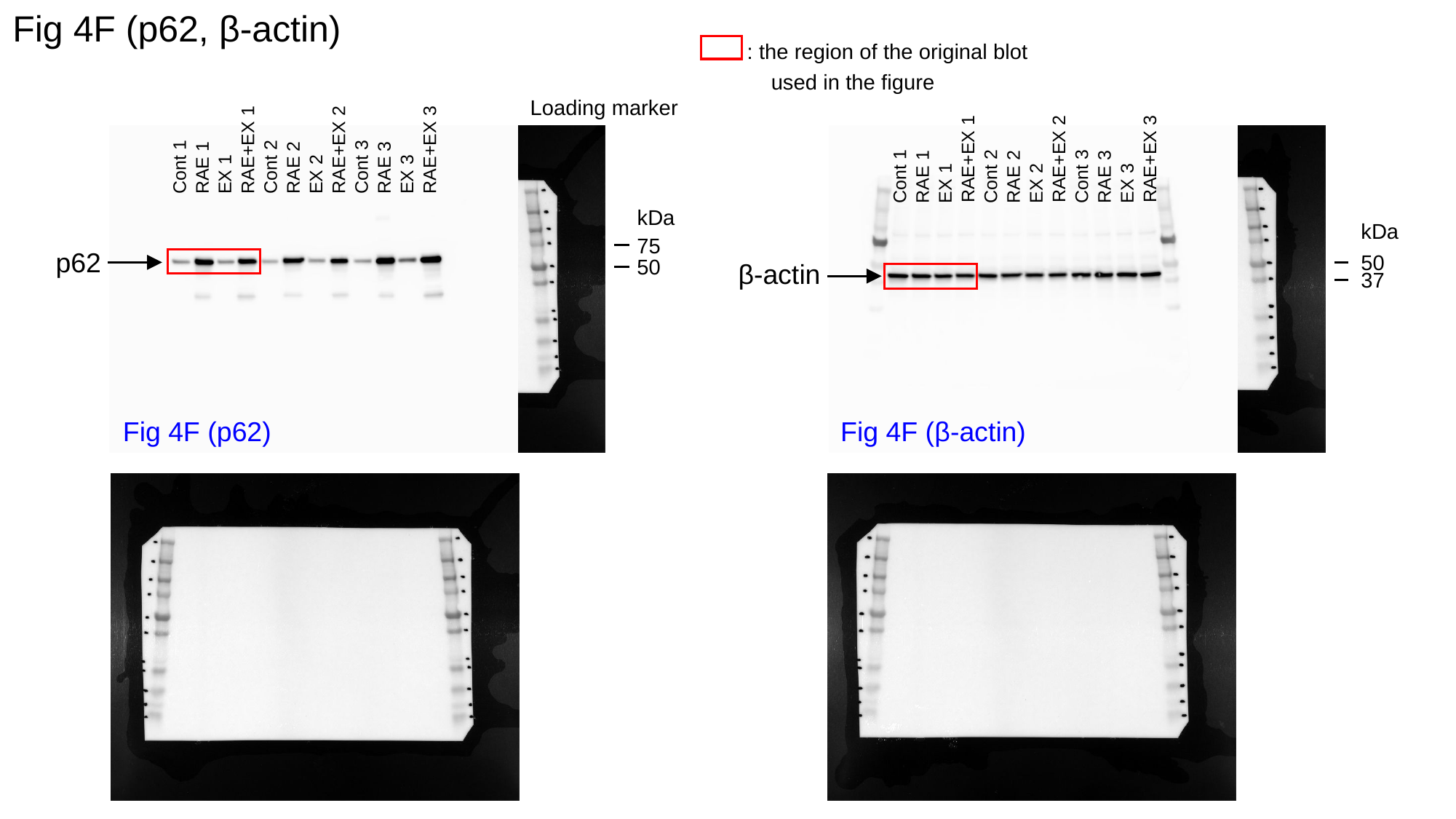

Fig 4F (p62, β-actin)
: the region of the original blot
 used in the figure
Loading marker
RAE+EX 1
RAE+EX 2
RAE+EX 3
Cont 1
Cont 2
Cont 3
RAE 1
RAE 2
RAE 3
EX 1
EX 2
EX 3
kDa
75
50
p62
Fig 4F (p62)
RAE+EX 1
RAE+EX 2
RAE+EX 3
Cont 1
Cont 2
Cont 3
RAE 1
RAE 2
RAE 3
EX 1
EX 2
EX 3
kDa
50
37
β-actin
Fig 4F (β-actin)

## Slide 19
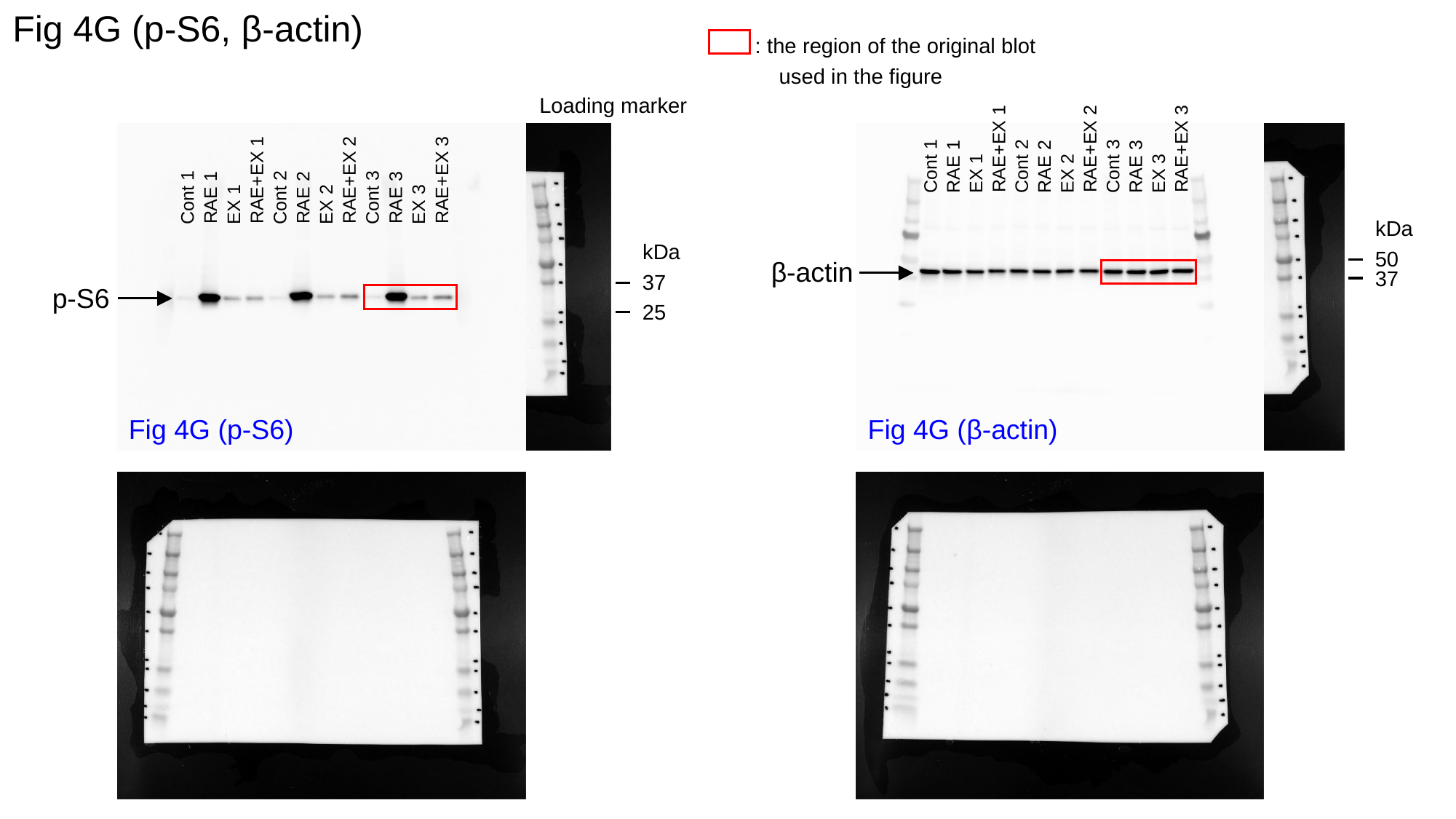

Fig 4G (p-S6, β-actin)
: the region of the original blot
 used in the figure
Loading marker
RAE+EX 1
RAE+EX 2
RAE+EX 3
Cont 1
Cont 2
Cont 3
RAE 1
RAE 2
RAE 3
EX 1
EX 2
EX 3
kDa
37
25
p-S6
Fig 4G (p-S6)
RAE+EX 1
RAE+EX 2
RAE+EX 3
Cont 1
Cont 2
Cont 3
RAE 1
RAE 2
RAE 3
EX 1
EX 2
EX 3
kDa
50
37
β-actin
Fig 4G (β-actin)

## Slide 20
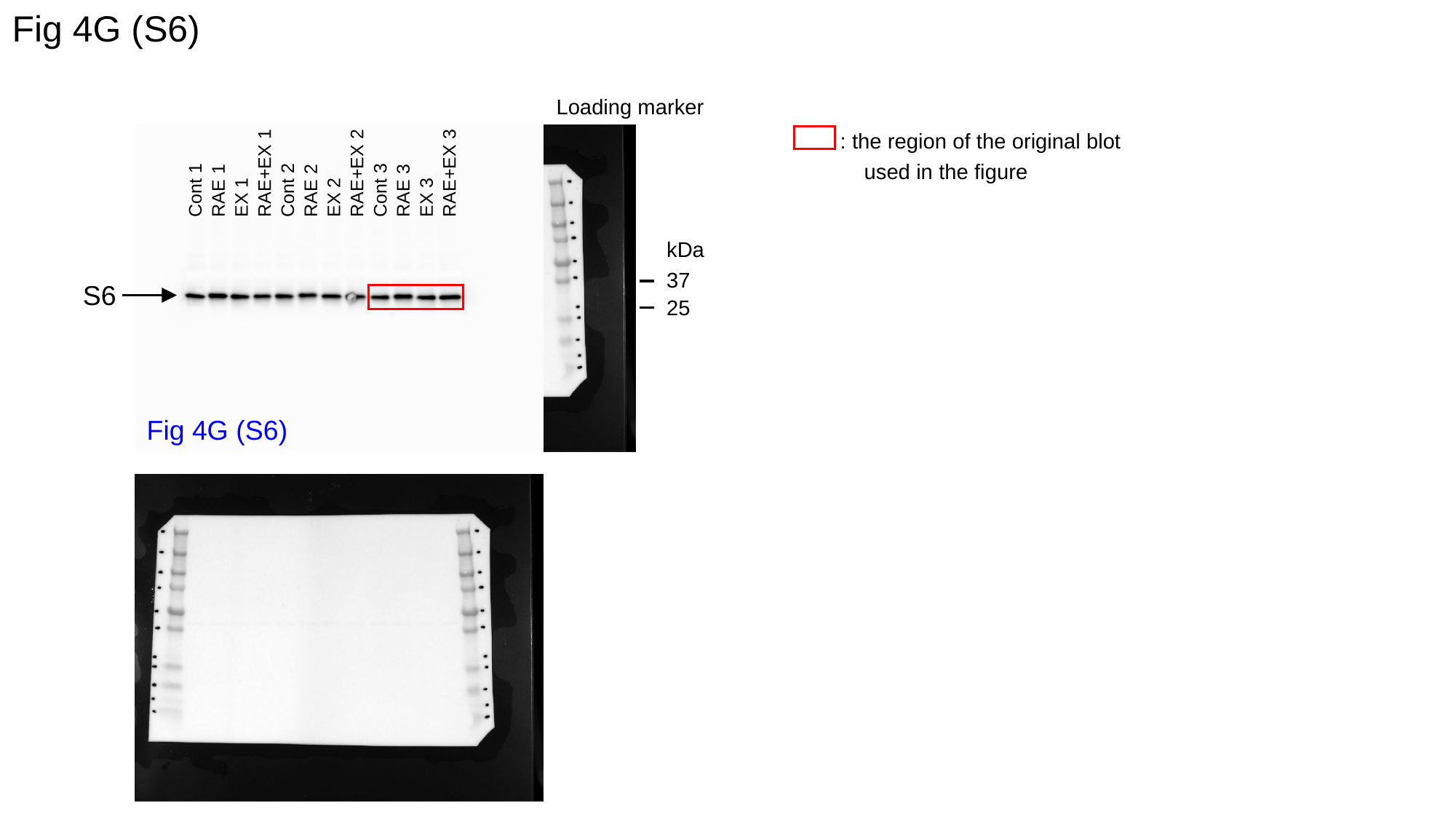

Fig 4G (S6)
Loading marker
RAE+EX 1
RAE+EX 2
RAE+EX 3
Cont 1
Cont 2
Cont 3
RAE 1
RAE 2
RAE 3
EX 1
EX 2
EX 3
kDa
37
25
S6
Fig 4G (S6)
: the region of the original blot
 used in the figure

## Slide 21
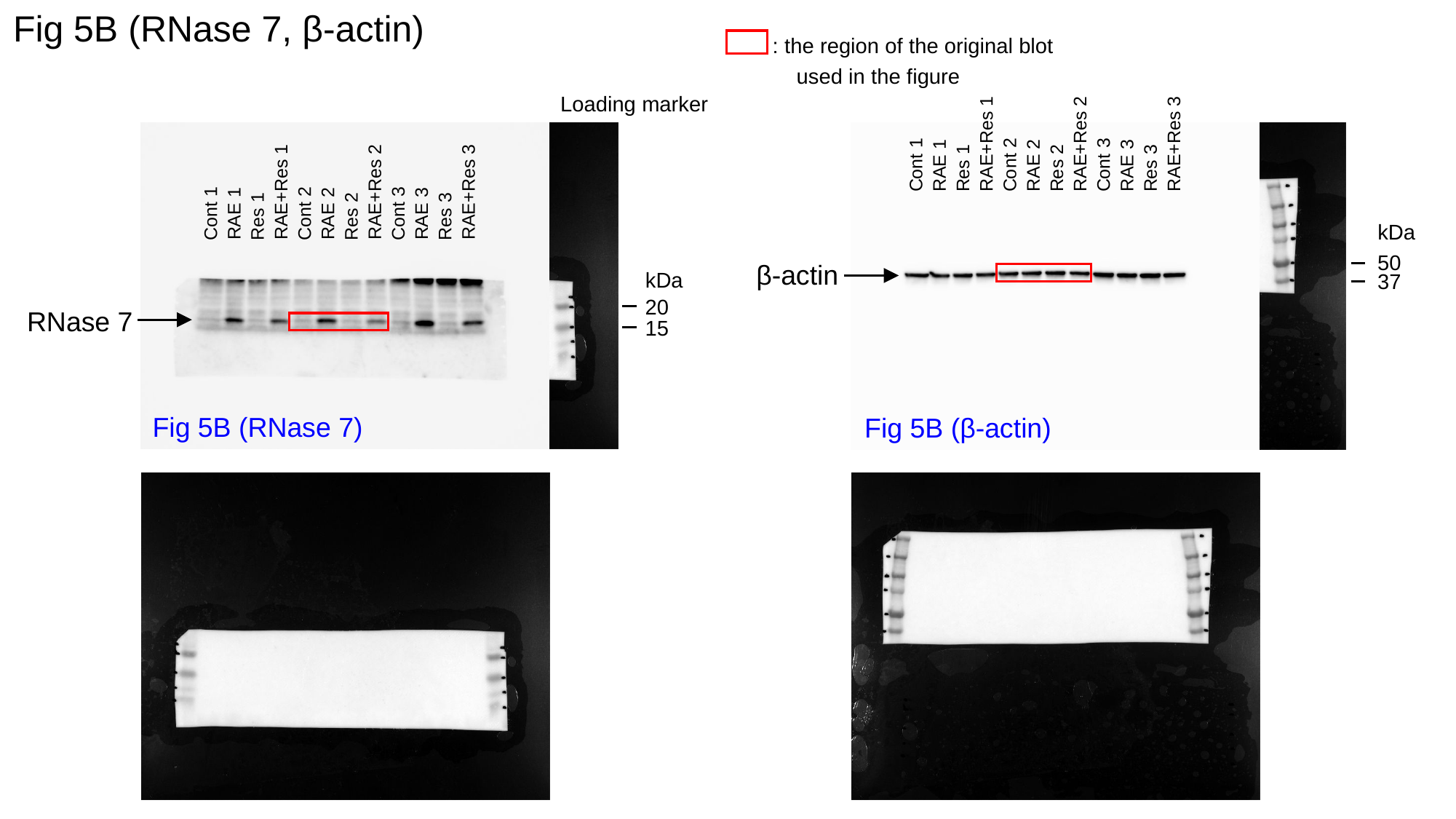

Fig 5B (RNase 7, β-actin)
: the region of the original blot
 used in the figure
RAE+Res 1
RAE+Res 2
RAE+Res 3
Cont 1
Cont 2
Cont 3
RAE 1
RAE 2
RAE 3
Res 1
Res 2
Res 3
kDa
50
37
β-actin
Fig 5B (β-actin)
Loading marker
RAE+Res 1
RAE+Res 2
RAE+Res 3
Cont 1
Cont 2
Cont 3
RAE 1
RAE 2
RAE 3
Res 1
Res 2
Res 3
kDa
20
15
RNase 7
Fig 5B (RNase 7)

## Slide 22
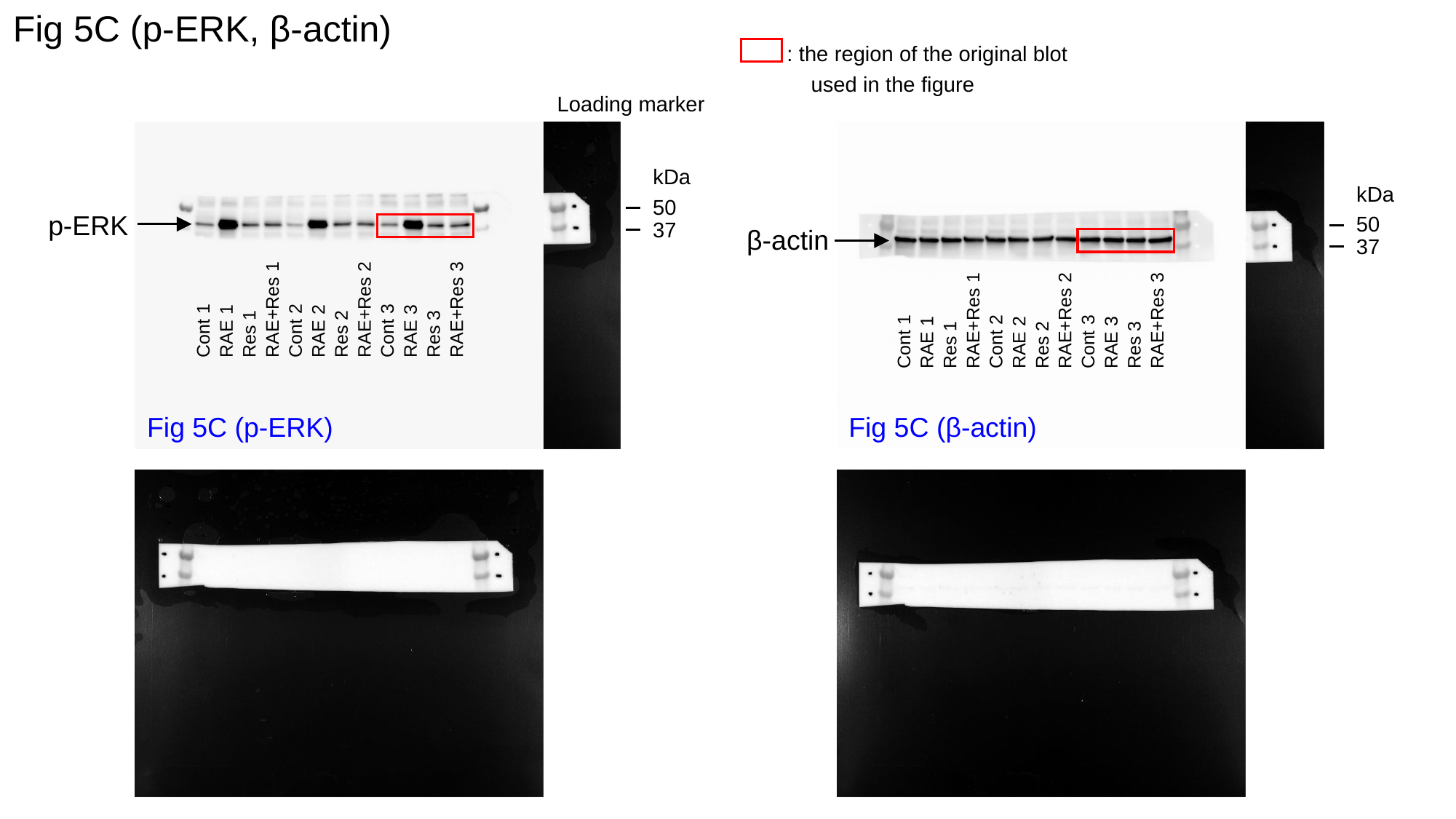

Fig 5C (p-ERK, β-actin)
: the region of the original blot
 used in the figure
Loading marker
kDa
50
37
p-ERK
RAE+Res 1
RAE+Res 2
RAE+Res 3
Cont 1
Cont 2
Cont 3
RAE 1
RAE 2
RAE 3
Res 1
Res 2
Res 3
Fig 5C (p-ERK)
kDa
50
37
β-actin
RAE+Res 1
RAE+Res 2
RAE+Res 3
Cont 1
Cont 2
Cont 3
RAE 1
RAE 2
RAE 3
Res 1
Res 2
Res 3
Fig 5C (β-actin)

## Slide 23
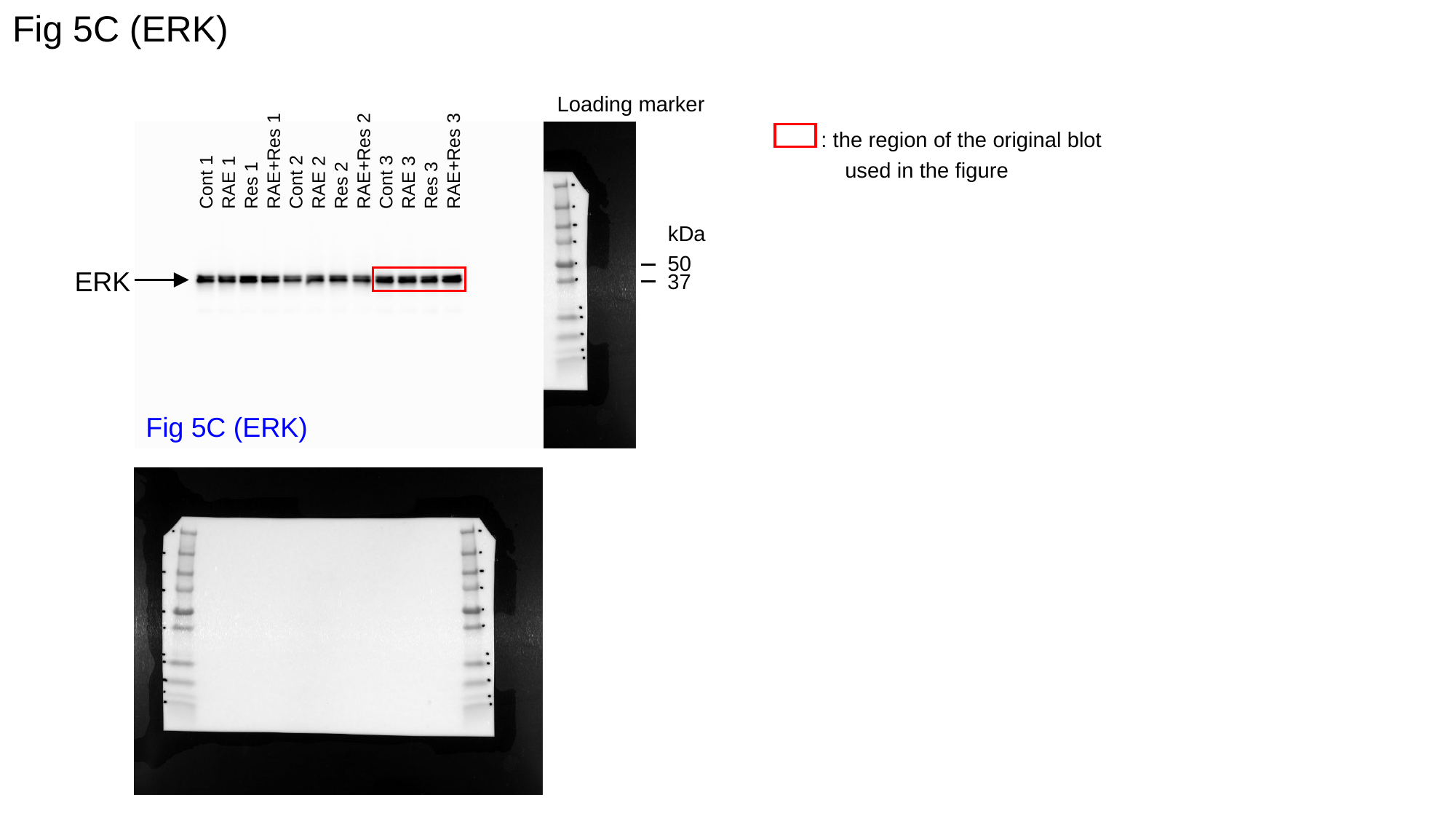

Fig 5C (ERK)
Loading marker
RAE+Res 1
RAE+Res 2
RAE+Res 3
Cont 1
Cont 2
Cont 3
RAE 1
RAE 2
RAE 3
Res 1
Res 2
Res 3
kDa
50
37
ERK
Fig 5C (ERK)
: the region of the original blot
 used in the figure

## Slide 24
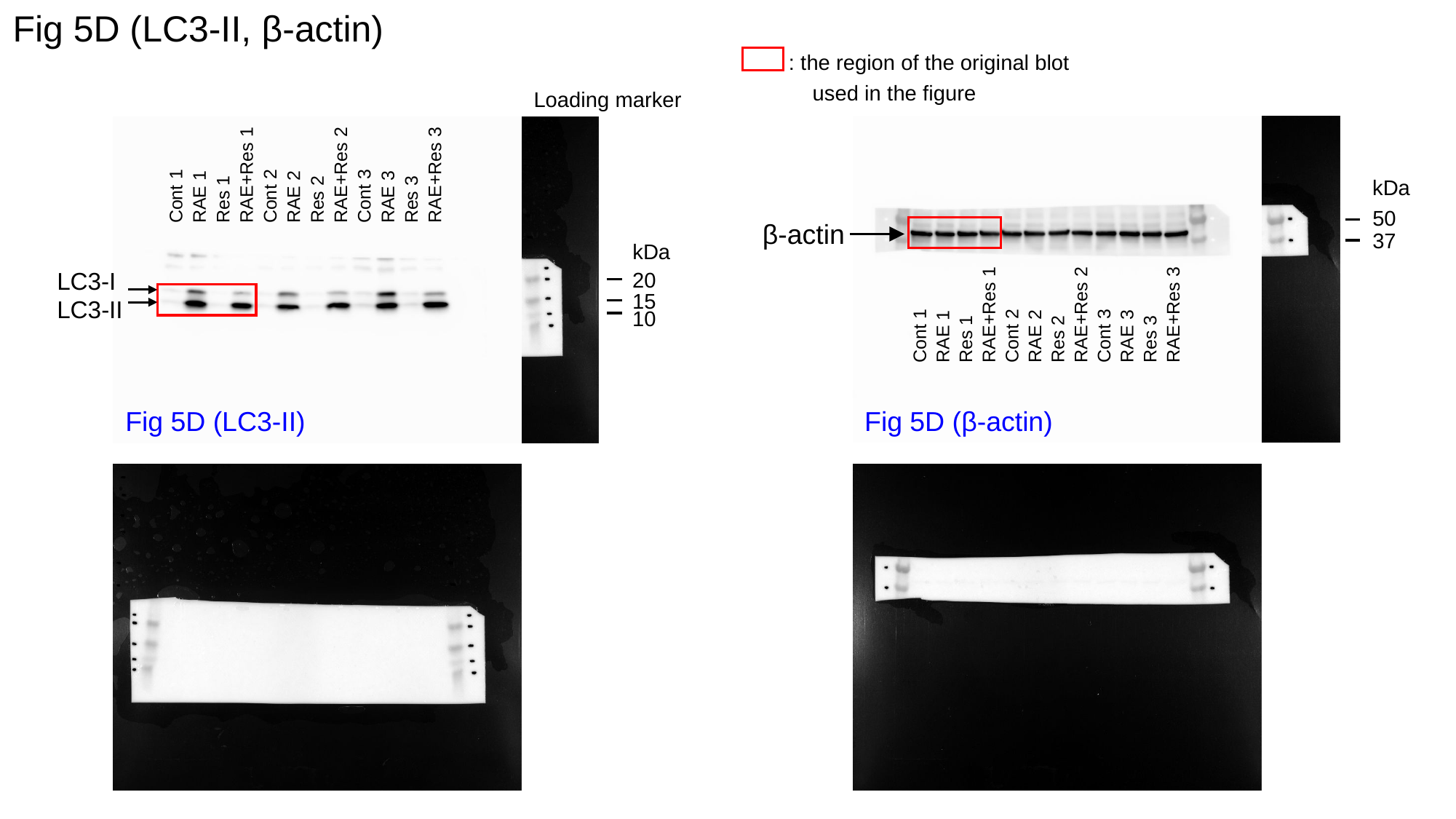

Fig 5D (LC3-II, β-actin)
: the region of the original blot
 used in the figure
Loading marker
RAE+Res 1
RAE+Res 2
RAE+Res 3
Cont 1
Cont 2
Cont 3
RAE 1
RAE 2
RAE 3
Res 1
Res 2
Res 3
kDa
20
15
10
LC3-I
LC3-II
Fig 5D (LC3-II)
kDa
50
37
β-actin
RAE+Res 1
RAE+Res 2
RAE+Res 3
Cont 1
Cont 2
Cont 3
RAE 1
RAE 2
RAE 3
Res 1
Res 2
Res 3
Fig 5D (β-actin)

## Slide 25
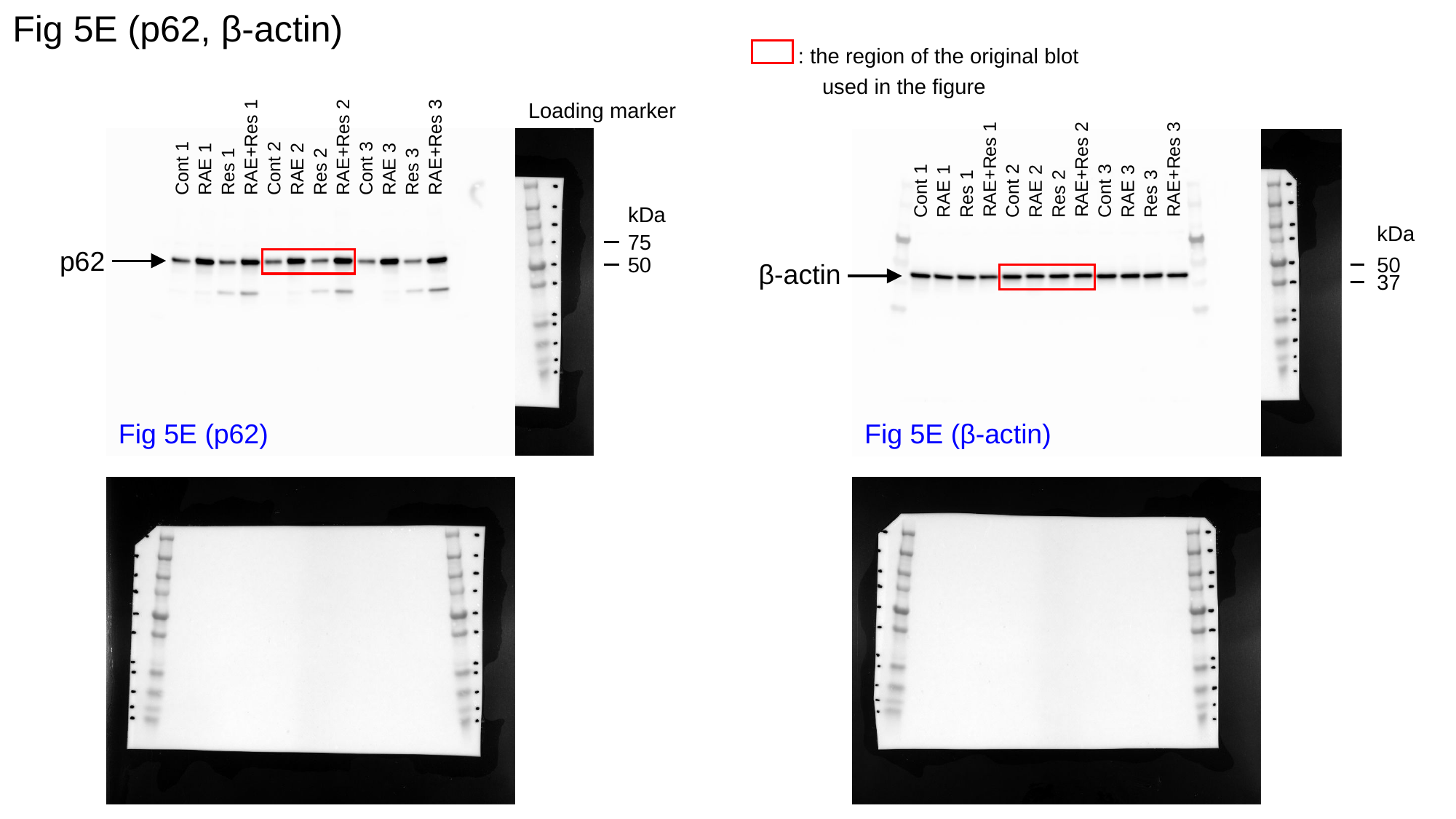

Fig 5E (p62, β-actin)
: the region of the original blot
 used in the figure
RAE+Res 1
RAE+Res 2
RAE+Res 3
Cont 1
Cont 2
Cont 3
RAE 1
RAE 2
RAE 3
Res 1
Res 2
Res 3
Loading marker
kDa
75
50
p62
Fig 5E (p62)
RAE+Res 1
RAE+Res 2
RAE+Res 3
Cont 1
Cont 2
Cont 3
RAE 1
RAE 2
RAE 3
Res 1
Res 2
Res 3
kDa
50
37
β-actin
Fig 5E (β-actin)

## Slide 26
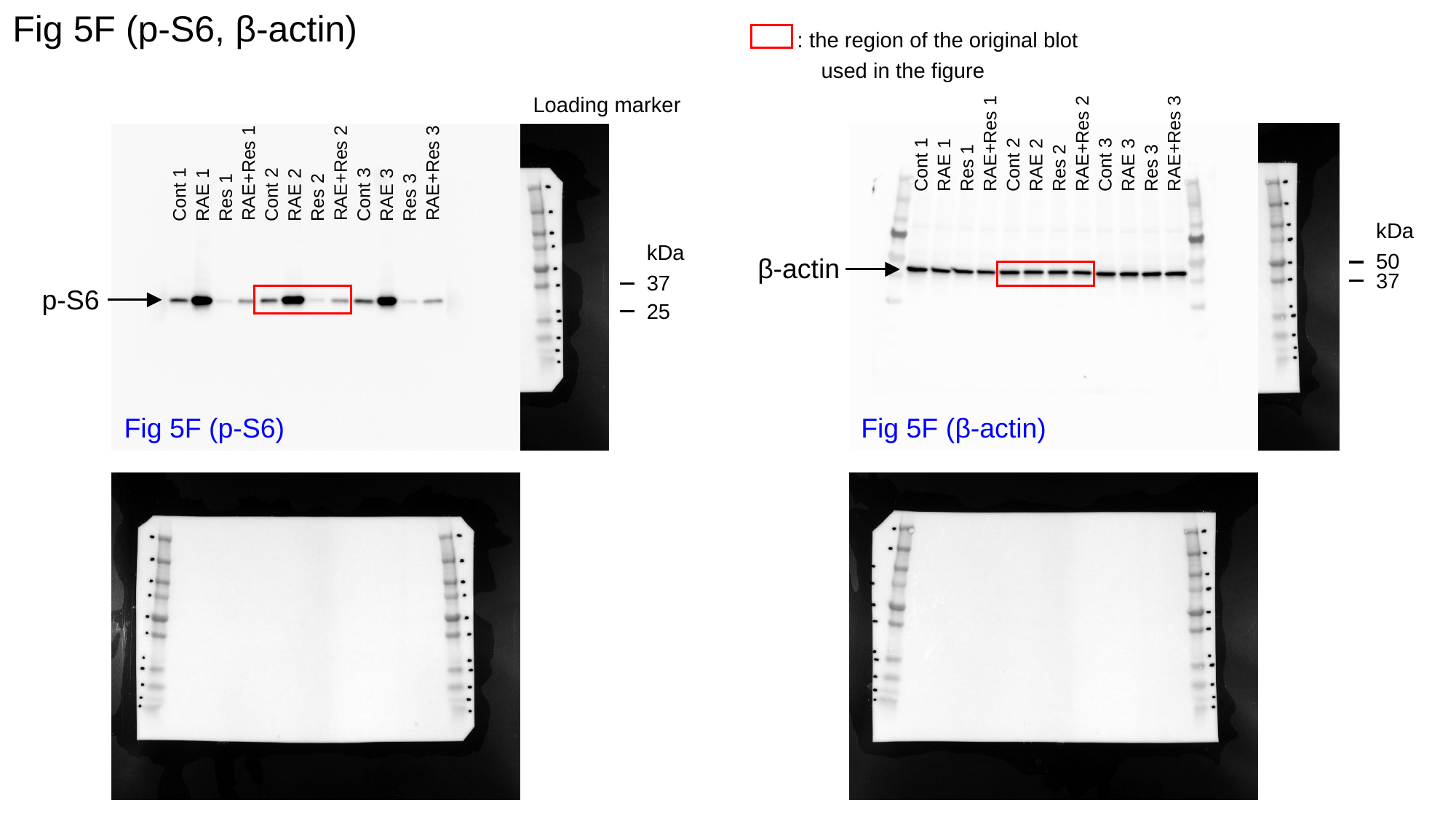

Fig 5F (p-S6, β-actin)
: the region of the original blot
 used in the figure
RAE+Res 1
RAE+Res 2
RAE+Res 3
Cont 1
Cont 2
Cont 3
RAE 1
RAE 2
RAE 3
Res 1
Res 2
Res 3
kDa
50
37
β-actin
Fig 5F (β-actin)
Loading marker
RAE+Res 1
RAE+Res 2
RAE+Res 3
Cont 1
Cont 2
Cont 3
RAE 1
RAE 2
RAE 3
Res 1
Res 2
Res 3
kDa
37
25
p-S6
Fig 5F (p-S6)

## Slide 27
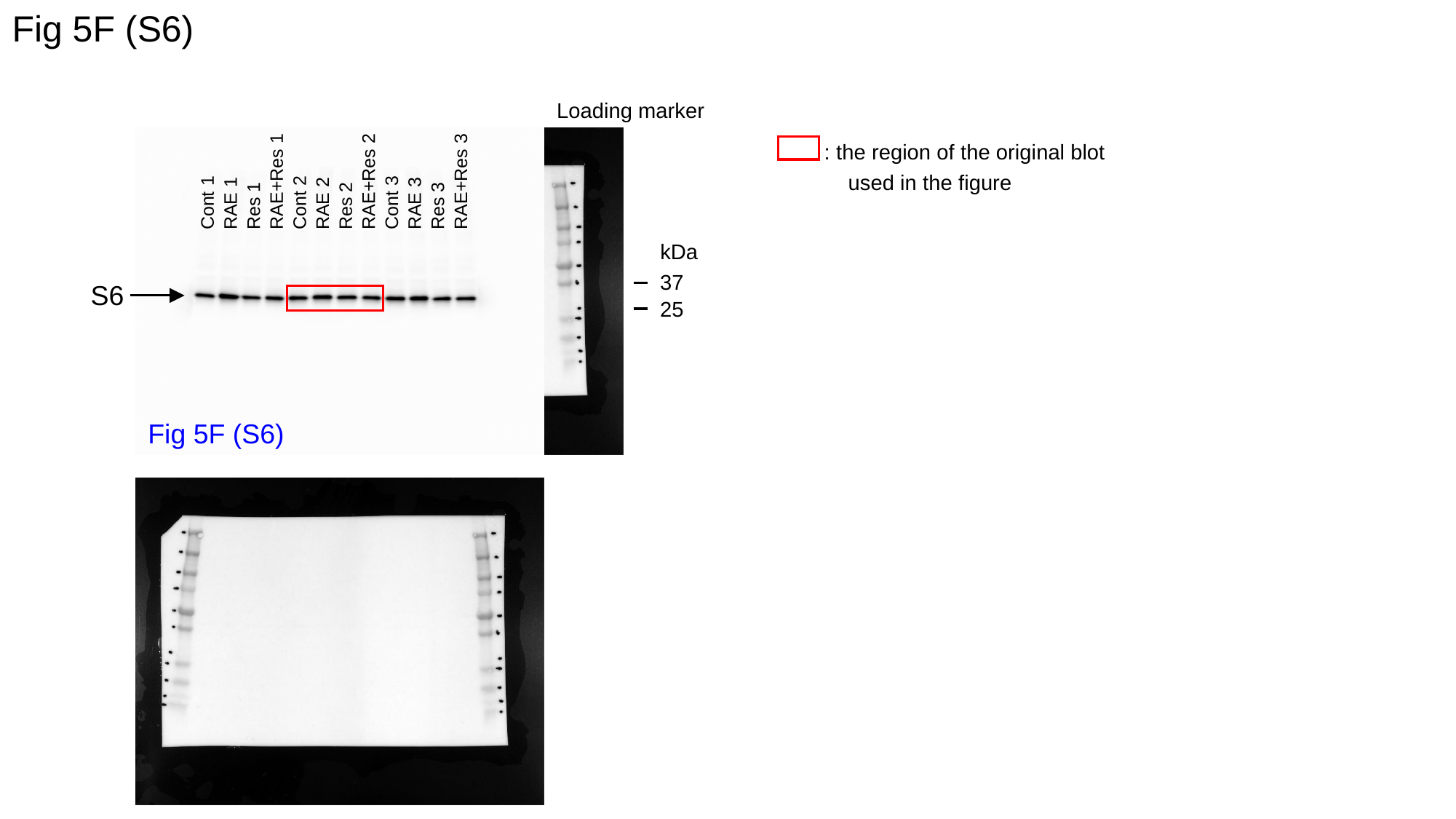

Fig 5F (S6)
Loading marker
RAE+Res 1
RAE+Res 2
RAE+Res 3
Cont 1
Cont 2
Cont 3
RAE 1
RAE 2
RAE 3
Res 1
Res 2
Res 3
kDa
37
25
S6
Fig 5F (S6)
: the region of the original blot
 used in the figure

## Slide 28
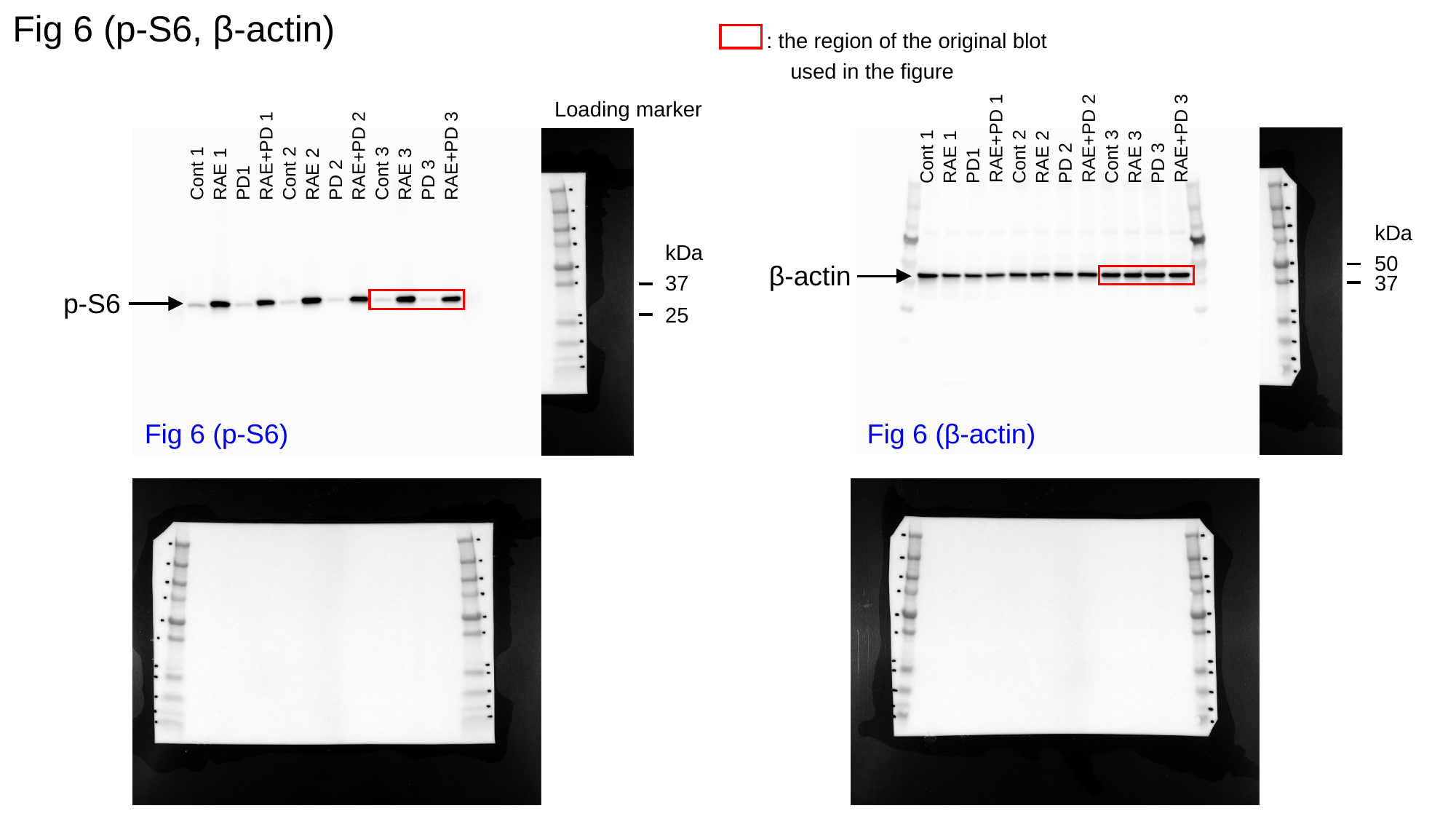

Fig 6 (p-S6, β-actin)
: the region of the original blot
 used in the figure
RAE+PD 1
RAE+PD 2
RAE+PD 3
Cont 2
Cont 3
Cont 1
RAE 1
RAE 2
RAE 3
PD 2
PD 3
PD1
kDa
50
37
β-actin
Fig 6 (β-actin)
Loading marker
RAE+PD 1
RAE+PD 2
RAE+PD 3
Cont 2
Cont 3
Cont 1
RAE 1
RAE 2
RAE 3
PD 2
PD 3
PD1
kDa
37
25
p-S6
Fig 6 (p-S6)

## Slide 29
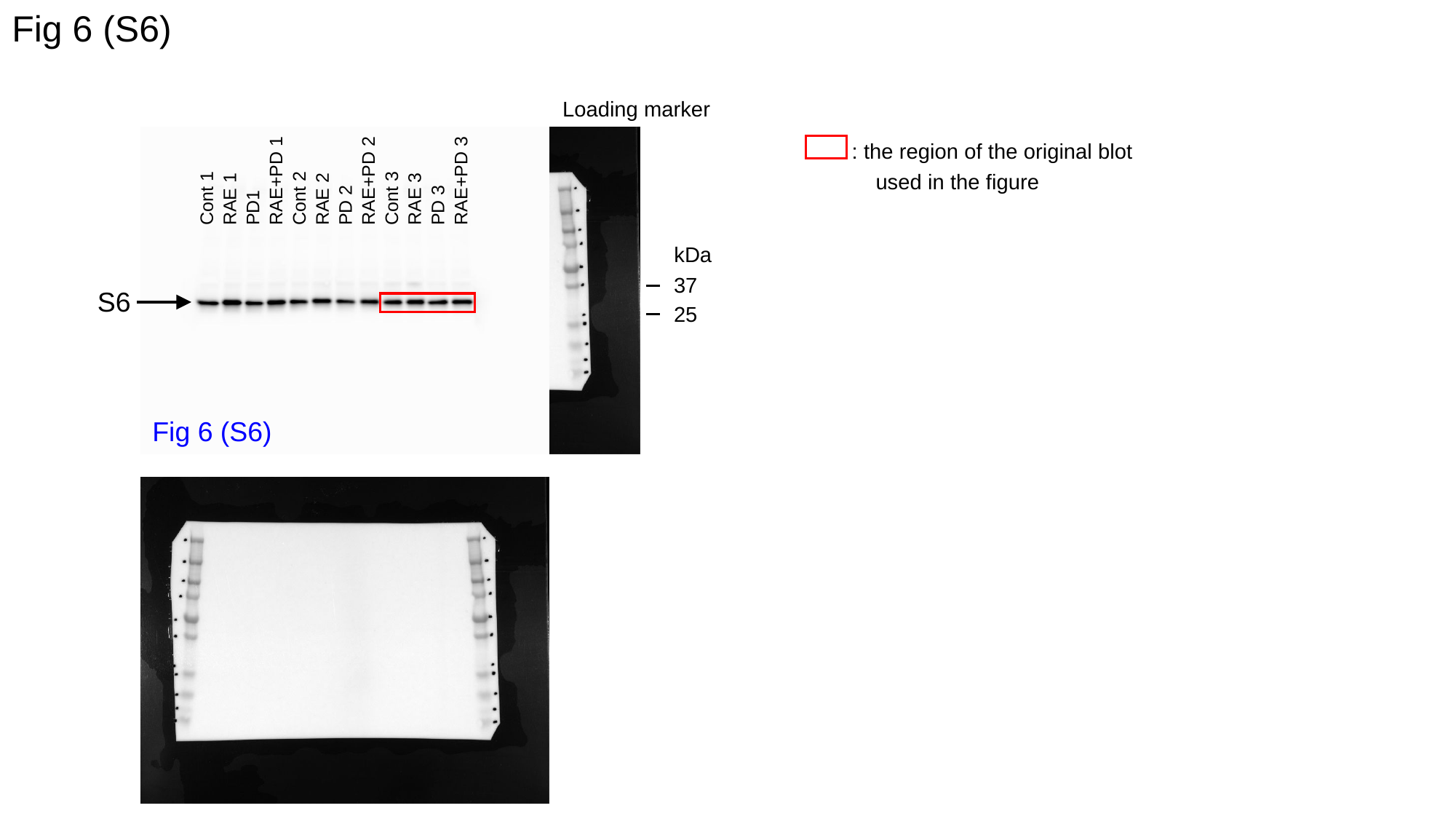

Fig 6 (S6)
Loading marker
RAE+PD 1
RAE+PD 2
RAE+PD 3
Cont 2
Cont 3
Cont 1
RAE 1
RAE 2
RAE 3
PD 2
PD 3
PD1
kDa
37
25
S6
Fig 6 (S6)
: the region of the original blot
 used in the figure

## Slide 30
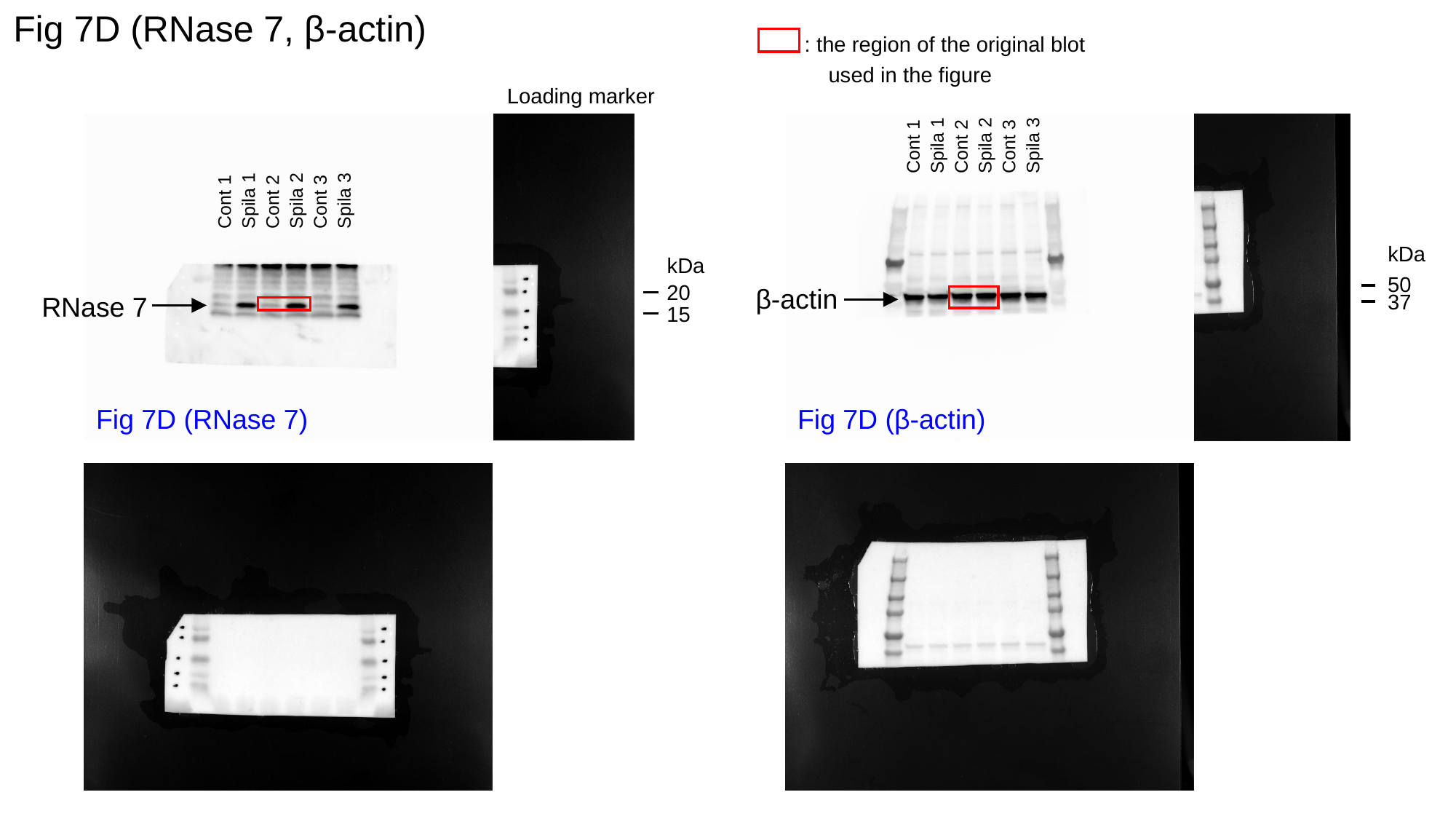

Fig 7D (RNase 7, β-actin)
: the region of the original blot
 used in the figure
Loading marker
Spila 2
Spila 3
Spila 1
Cont 3
Cont 1
Cont 2
kDa
20
15
RNase 7
Fig 7D (RNase 7)
Spila 2
Spila 3
Spila 1
Cont 3
Cont 1
Cont 2
kDa
50
37
β-actin
Fig 7D (β-actin)

## Slide 31
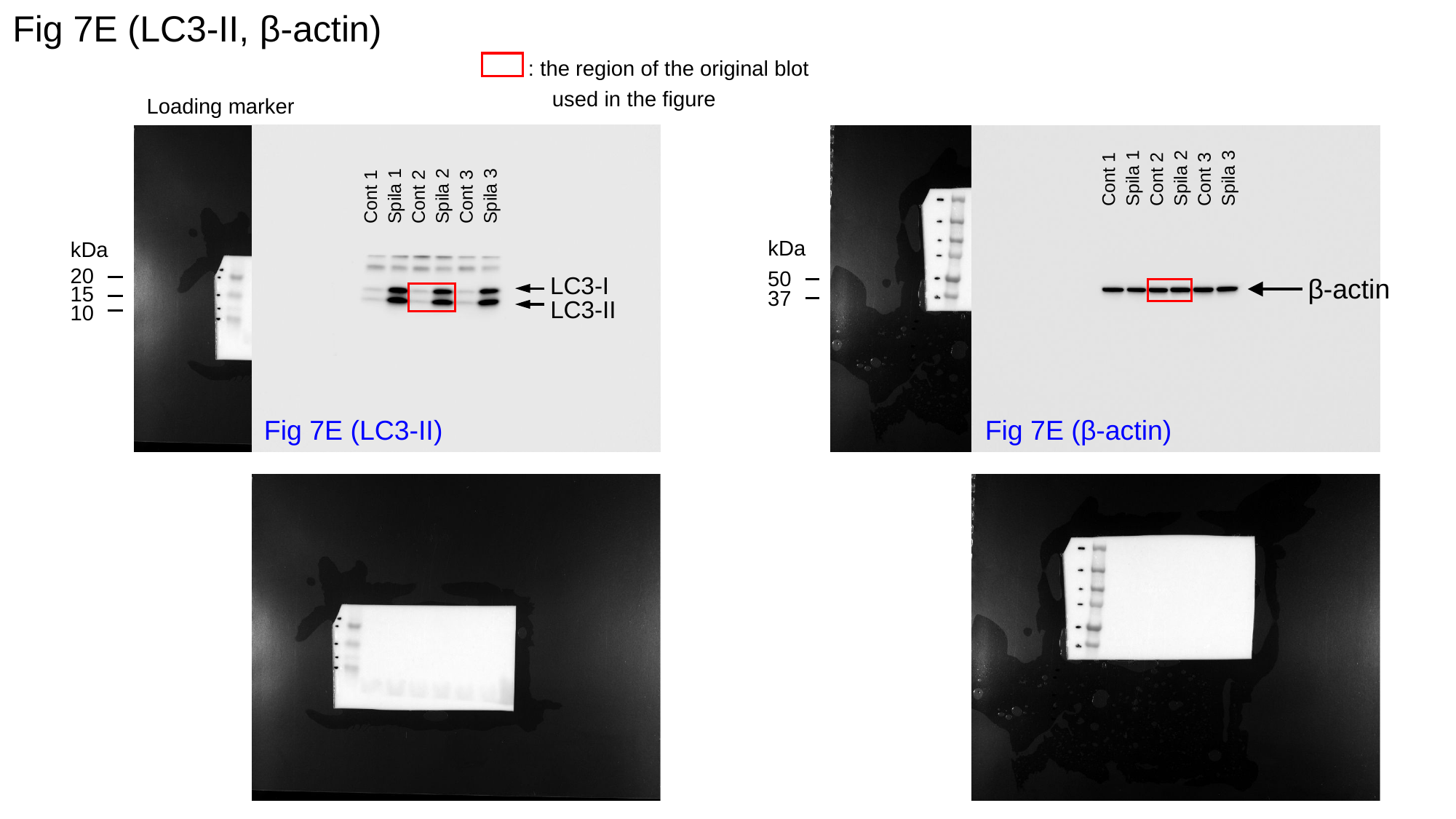

Fig 7E (LC3-II, β-actin)
: the region of the original blot
 used in the figure
Loading marker
Spila 2
Spila 3
Spila 1
Cont 3
Cont 1
Cont 2
kDa
20
15
10
LC3-I
LC3-II
Fig 7E (LC3-II)
Spila 2
Spila 3
Spila 1
Cont 3
Cont 1
Cont 2
kDa
50
37
β-actin
Fig 7E (β-actin)

## Slide 32
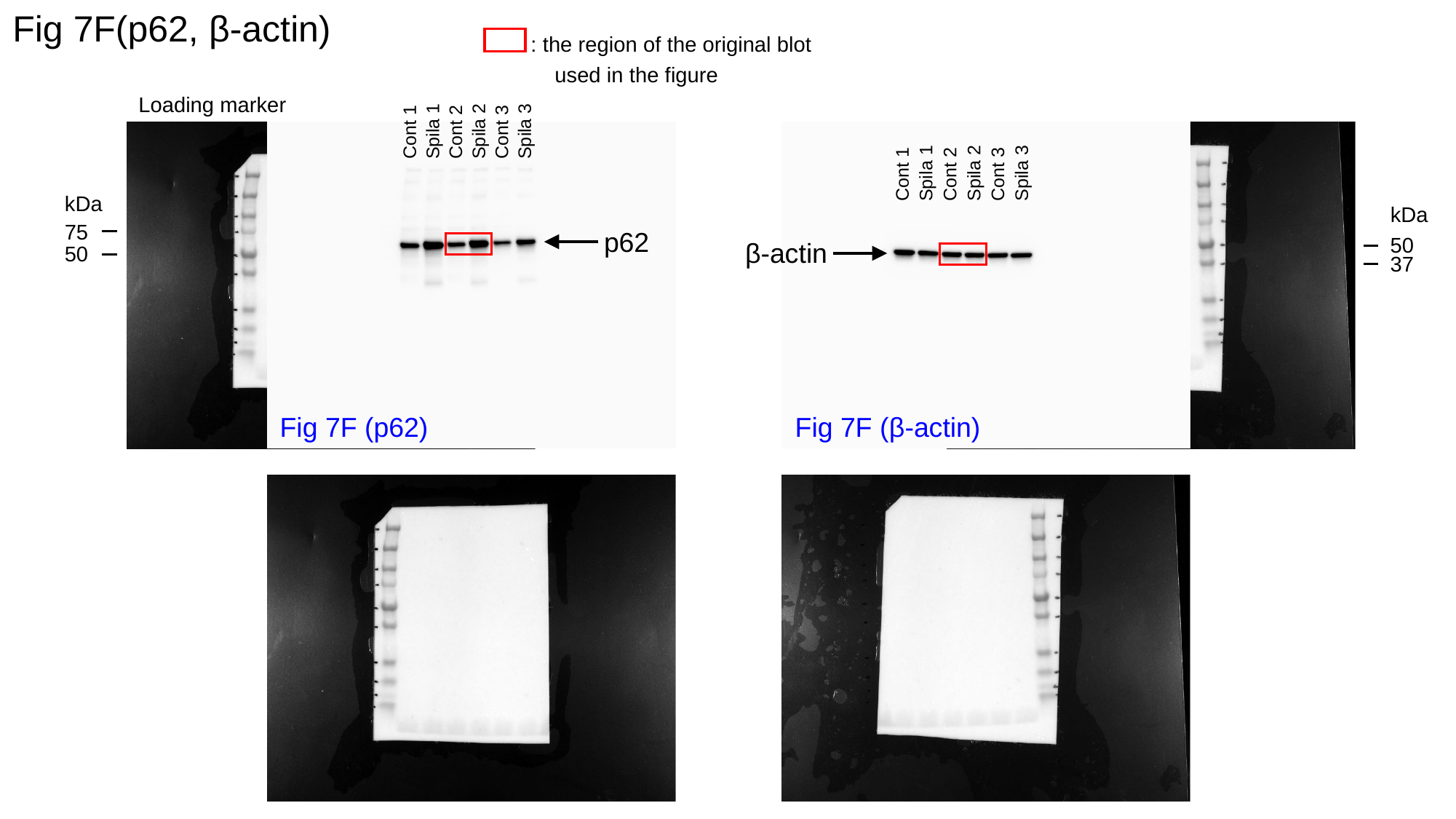

Fig 7F(p62, β-actin)
: the region of the original blot
 used in the figure
Loading marker
Spila 2
Spila 3
Spila 1
Cont 3
Cont 1
Cont 2
kDa
75
50
p62
Fig 7F (p62)
Spila 2
Spila 3
Spila 1
Cont 3
Cont 1
Cont 2
kDa
50
37
β-actin
Fig 7F (β-actin)

## Slide 33
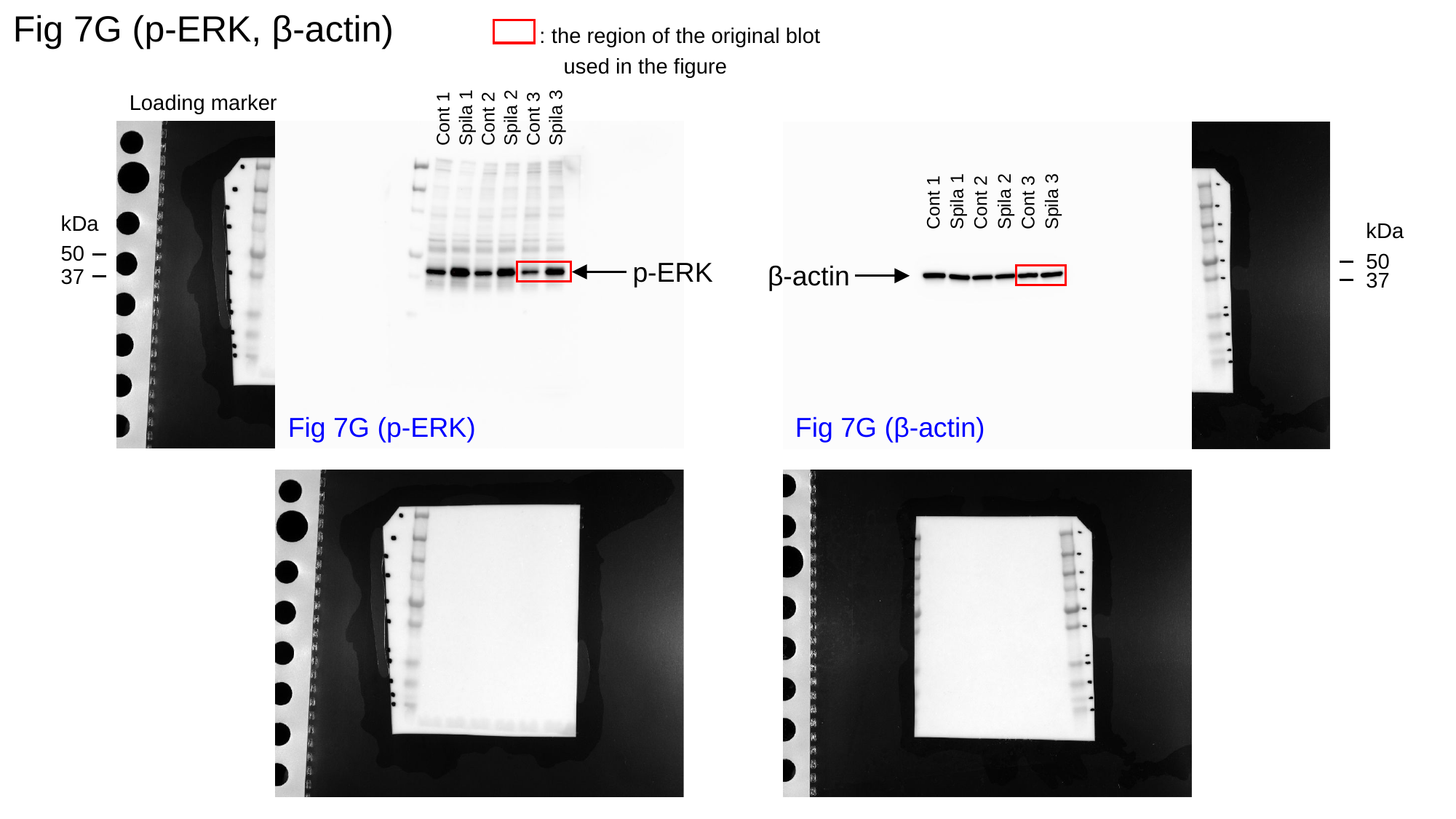

Fig 7G (p-ERK, β-actin)
: the region of the original blot
 used in the figure
Spila 2
Spila 3
Spila 1
Cont 3
Cont 1
Cont 2
Loading marker
kDa
50
37
p-ERK
Fig 7G (p-ERK)
Spila 2
Spila 3
Spila 1
Cont 3
Cont 1
Cont 2
kDa
50
37
β-actin
Fig 7G (β-actin)

## Slide 34
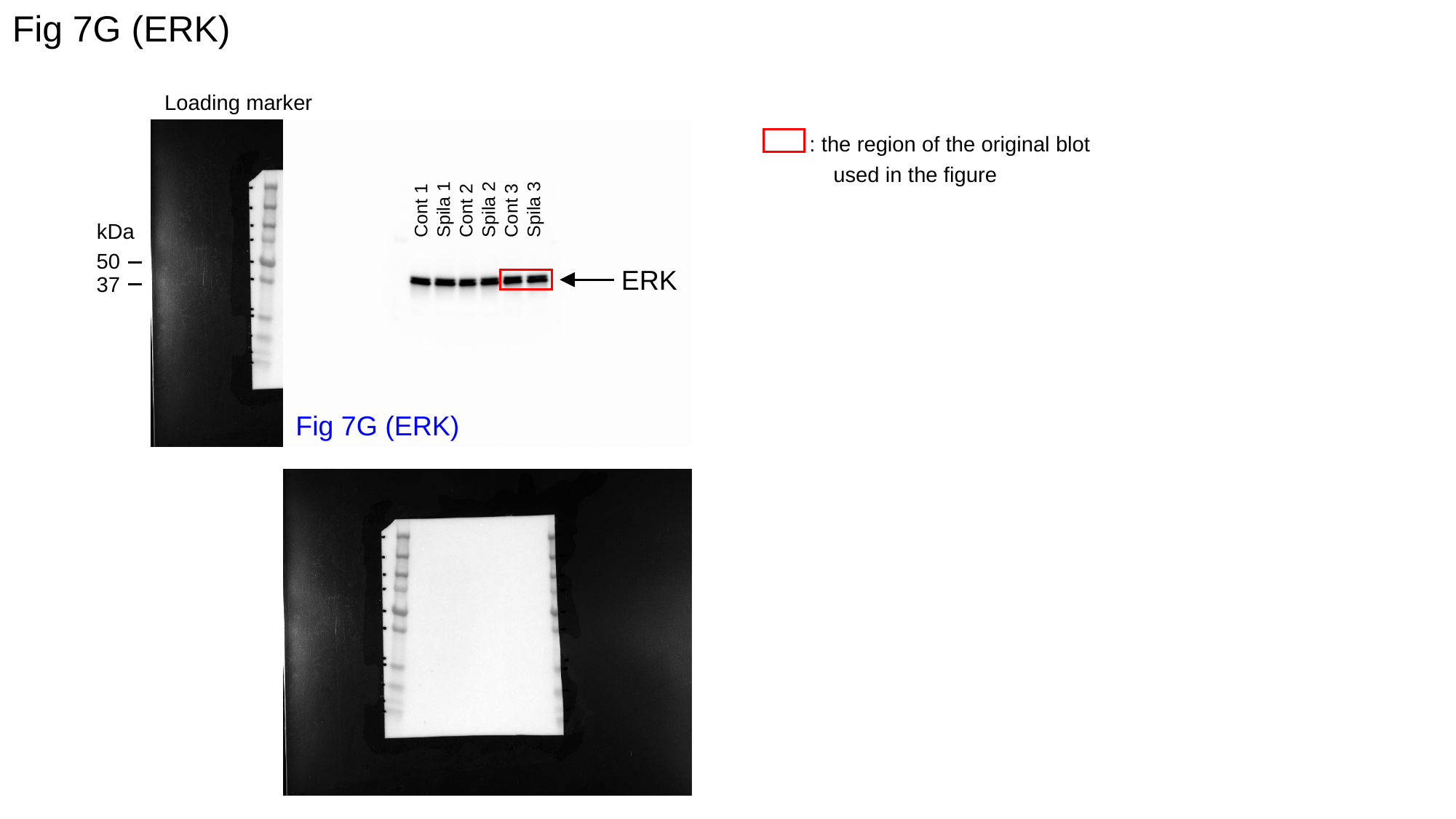

Fig 7G (ERK)
Loading marker
Spila 2
Spila 3
Spila 1
Cont 3
Cont 1
Cont 2
kDa
50
37
ERK
Fig 7G (ERK)
: the region of the original blot
 used in the figure
