# Supplementary material for: Ruscus aculeatus extract promotes RNase 7 expression through ERK activation following inhibition of late-phase autophagy in primary human keratinocytes
Source: PLoS One. 2024 Dec 3;19(12):e0314873. doi: 10.1371/journal.pone.0314873 (PMC11614269; doi:10.1371/journal.pone.0314873)
Supplement: Attachment — Submitted filename: Response to Reviewers 202407.docx [file pone.0314873.s003.docx]

**Response to Reviewers**

We wish to express our appreciation for the insightful comments that we received from the reviewers. We feel that the comments have helped us to significantly improve our paper. Our responses to each of the reviewers’ comments are highlighted in red both here and in our revised manuscripts. Additionally, based on the reviewer’s comments, we have voluntarily modified some text, which is highlighted in blue in the revised manuscript.

**Reviewer #1**

1) *Fig3D: LC3 punctae are not visible in the figures. To clarify the claim of*

*early and late phase autophagy authors are requested to provide mRFP-GFPLC3 data.
Authors should provide the better images or repeat with LC3 and p62 punctae.*

[Our response]

We have accepted your suggestion regarding the image quality of Fig 3D. Thus, to clarify the colocalization of LC3 and p62, we adjusted the resolution (quality) of Fig 3D. Please check the revisions to Fig 3D (Fig 3E in the revised Fig 3). Translating figures from TIFF images into PDF seems to degrade image quality. Therefore, the revised figure of the TIFF image (Fig 3E) is displayed here (preferably, check the TIFF image itself).

As you pointed out, the method using mRFP-GTP-LC3 is effective as one of the methods to examine the autophagy status. In the current study, we investigated the autophagy status with the flux assay by chloroquine, a late-phase autophagy inhibitor, the increase in protein expression levels of p62, and the colocalization of LC3 and p62 using immunofluorescence analysis. These methods are effective as a way to examine the autophagy status under certain conditions, as is the mREP-GTP-LC3 method. Our results strongly suggest that RAE inhibits the late-phase autophagy and that autophagosomes are accumulated by RAE treatment. Therefore, we believe that applying mRFP-GTP-LC3 is not essential to our study.

2) *Fig 4A: authors are requested to provide the immunoblots to prove the*

*points with wortmanin like provided with EX527, because here authors are claiming to show the outcome of inhibition of early and late phase autophagy on RNAase 7 expression including p-ERK, also authors are requested to provide the evidence of early autophagy genes ULK1 and PIK3C3 KD and the effect on all the proteins LC3, p62, p-ERK and RNAase7, similary authors are requested to conduct late phase autophagy gene KD ATG7 which effects LC3 lipidation and provide the evidence for the effect on RNAase7.*

[Our response]

First, we performed an exploratory experiment using wortmannin to determine whether early-phase autophagy (basal autophagic activity) based on the mTOR-dependent autophagy pathway is involved in RAE-induced RNase 7 expression. Accordingly, we evaluated only the mRNA expression of RNase 7. Since RAE-induced RNase 7 expression was inhibited by wortmannin, we nest investigated in detail the mechanisms in which the early-phase autophagy is involved, using EX-527, an inhibitor of SIRT1, and resveratrol, an activator of SIRT1. In experiments with EX-527 and resveratrol, mRNA expression of RNase 7 and protein expression of RNase 7, p-ERK, LC3-II and p62 were examined. We indicated that in the EX-527 experiment, EX-527 suppressed the early-phase autophagy (basal autophagic activity) by showing that p-S6 expression in the presence of EX-527 was increased compared with control (control vs. EX or RAE+EX in Fig 4D at the first submission). Further, we exhibited that in the resveratrol experiment, resveratrol accelerated the early-phase autophagy by showing that p-S6 expression in the presence of resveratrol was decreased compared with control (control vs. Res or RAE+Res in Fig 5C at the first submission). These results, together with the results of the flux assay, p62 western blot, and immunofluorescence analysis clearly, and logically suggest that early-phase autophagy (basal autophagic activity) and inhibition of late-phase autophagy are required for RAE-induced RNase 7 expression. As mentioned above, the aim of our mechanism study is to indicate how the early-phase autophagy and the late-phase autophagy, namely autophagy itself, participate in RAE-induced RNase 7 expression. Therefore, we believe that the examination of protein expression of RNase 7, p-ERK, LC3-II, and p62 in the wortmannin experiment (Fig 4A), and knockdown experiments for ULK1, PI3K, and ATG7 are not essential. For reference, at present, we consider the mechanisms of RAE-induced RNase 7 expression as shown in the following figure.

However, the mechanisms are not completely elucidated. Further studies including knockdown experiments of genes such as ATG7 will be needed to clarify the mechanisms. Since this point is considered to be important, we have corrected the text as follows.

(page 23, lines 543–546 in our revised manuscript)

The mechanism by which RAE induces RNase 7 expression was not fully elucidated in this study. Therefore, further studies, including knockdown experiments of genes such as ULK1, ATG7, and SIRT1, will be needed to better understand RNase 7 expression induced by RAE treatment.

3) *Fig 4: Authors have mentioned EX-527 as mTOR activators although*

*literature search shows ERX as mTOR suppressor (which means autophagy activator), authors are requested to clarify and rectify the claim here, otherwise it does not conclude what authors are trying to show here. Authors are claiming RAE as late phase autophagy inhibitor but p-S6 show it is activator or mTOR which controls the early autophagy, therefore authors own results reject the claim of inhibition of late phase autophagy, although it can be seen autophagy regulates the expression of RNAase7 expression.*

[Our response]

EX-527 is well known to be a specific SIRT1 inhibitor; numerous reports have shown that EX-527 inhibits autophagy via inhibition of SIRT1 receptor^*1–3^. Therefore, as shown in our manuscript, EX-527 is an autophagy suppressor, not an activator. We believe that our experimental theory is consistent.

*1: Yang X et al. SIRT1 inhibition promotes atherosclerosis through impaired autophagy. Oncotarget. 2017; 8(31):51447–51461.

*2: Luo G et al. Sirt1 promotes autophagy and inhibits apoptosis to protect cardiomyocytes from hypoxic stress. Int J Mol Med. 2019; 43(5):2033–2043.

*3: Wu Y et al. Suppression of sirtuin 1 alleviates airway inflammation through mTOR-mediated autophagy. Mol Med Rep. 2020; 22(3):2219–2226.

Accordingly, we have corrected the text as follows.

(page 15, lines 349–350 in our revised manuscript)

In addition, numerous reports have shown that EX-527 inhibits autophagy via inhibition of the SIRT1 receptor [34–36].

RAE activated S6. As you pointed out, in general, the activation of S6 (increase in protein expression levels of p-S6) suggests the activation of mTOR. However, RAE-induced RNase 7 expression required early-phase autophagy (basal autophagic activity), implying that mTOR is not activated in RNase 7 expression induced by RAE treatment (Fig 4 and 5). In addition, we showed that S6 is not a downstream target of ERK (Fig 6). These findings suggest that S6 is not the direct factor in the pathway via which RAE induces RNase 7 expression; this was mentioned in the “Discussion” of our first manuscript (pages 22 and 23, lines 517–529 in our revised manuscript). Why S6 is activated in RAE-induced RNase 7 expression was also mentioned in the “Discussion” of our first manuscript (page 23, lines 530–546 in our revised manuscript). However, the conclusion that S6 is not the direct factor in the pathway via which RAE induces RNase 7 expression was not described in our first manuscript. We apologize for any misunderstanding we may have caused you. To make it easier for readers to understand the complex mechanisms, we have corrected the text as follows.

(page 23, lines 526–527 in our revised manuscript)

These findings suggest that S6 is not activated by the mTOR/S6K and/or ERK/RSK pathways; it seems that S6 is not the direct (key) factor in the pathway via which RAE induces RNase 7 expression.

4) *figure 6 alone with one immunoblot is not required, although reviewer understands it is different from other figures but to make it a separate figure more data is required.*

[Our response]

Thank you for your suggestion. We attempted to make adjustments, such as integrating Fig 6 into the other figures. However, we observed that it difficult to make such adjustments because it would cause discomfort in the overall structure and explanation flow of our paper. We believe that Fig 6 shows an independent and meaningful experiment to examine the mechanisms involved in RAE-induced RNase 7 expression. Although Fig 6 has only one graph, we would like to leave Fig 6 as it is. Several papers previously published in PLOS ONE with figures that have only one graph, as referenced below. We hope you will reconsider this issue.

1) Simanski M et al. IL-17A and IFN-γ synergistically induce RNase expression via STAT3 in primary keratinocytes. PLoS One. 2013; 8(3):e5931.

2) Firat YH et al. Infection of keratinocytes with Trichophytum rubrum induces epidermal growth factor-dependent RNase 7 and human beta-defensin-3 expression. PLoS One. 2014; 9(4):e93941.

3) Gonzalez-Curiel I et al. 1,25-Dihydroxyvitamin D3 induces LL-37 and HBD-2 production in keratinocytes from diabetic foot ulcers promoting wound healing: An in vitro model. PLoS One. 2014; 9(10):e111355.

*Minor comments:
1) authors are requested to keep blots for p62 and LC3 together in every figures.*

[Our response]

Thank you for your suggestion. Each western blot diagram of LC3 or p62 is intended to compare the difference in intensity between RAE and RAE+Res, respectively. Thus, we consider that they are placed in consideration of where necessary. We believe that the current situation is clear enough to readers. Could you please reconsider this issue?

*2) all the blots specifically where the difference is there as per authors claim*

[Our response]

We added the information shown below to the western blot diagrams in the “Results” section to show points of comparison for ease of understanding. Please check the revised “Results” section.

Example: (Fig 2D; RAE vs. RAE+PD98059)

Moreover, for ease of understanding, we have named each western blot diagram in Fig 4C and 5B.

In the case of Fig 4; RNase 7: Fig 4C, ERK: Fig 4D, LC3: Fig 4E, p62: Fig 4F, and S6: Fig 4G

In the case of Fig 5; RNase 7: Fig 5B, ERK: Fig 5C, LC3: Fig 5D, p62: Fig 5E, and S6: Fig 5F

Please check the revised Fig 4 and 5.

**Reviewer #2**

1. *Acknowledgment of Manuscript Quality:*

*"The manuscript was effectively written, with data presented in a clear and concise manner."*

[Our response]

Thank you for the good rating.

2. *Recognition of Experimental Design:*

*"The experimental design was robust, and the data appeared organized and well-presented."*

[Our response]

Thank you for the good rating.

3. *Appreciation for Statistical Analysis:*

*"I appreciate the author's use of valid statistical analysis tests, which bolster the credibility of the findings."*

[Our response]

Thank you for the good rating.

4. *Suggestion for Experimentation Expansion:*

*"While HCQ was appropriately used to inhibit phagolysosome formation, I recommend the exploration of additional late-phase autophagy inhibitors, such as Bafilomycin A1. This broader experimentation would enhance the comprehensiveness of the study."*

[Our response]

Thank you for your suggestion. It is well known that the inhibitory mechanisms by which HCQ and bafilomycin A1 inhibit late-phase autophagy are different. Therefore, as you pointed out, to enhance the comprehensiveness of our study, we performed an additional experiment with bafilomycin A1. As a result, we observed that in the experiment using bafilomycin A1 as well as HCQ, RAE inhibits late-phase autophagy. Since we had a new insight, we have corrected the related text as follows. Moreover, please check the revised Fig 3.

(pages 25 and 26, lines 589–601 in our revised manuscript)

In the autophagic flux assay with HCQ and BA1, we showed that RAE inhibited late-phase autophagy in RNase 7 expression induced by RAE treatment. The protein expression levels of LC3-Ⅱ induced by RAE in the presence of HCQ or BA1 were significantly decreased compared with those with RAE alone. This suggests that the inhibitory effect of RAE was weakened by them. In other word, it implies that the active compound (spilacleoside) in RAE inhibits late-phase autophagy at least in part in a manner similar to HCQ and BA1. Moreover, because the concentration of spilacleoside in RAE (83 nM) is much lower than HCQ (10 μM) and almost equal to BA1 (50 nM), its activity as a late-phase autophagy inhibitor might be quite high. Therefore, spilacleoside might become a novel lead compound for the development of highly effective autophagy inhibitors. It is well known that the mechanisms by which HCQ [50] and BA1 [51] inhibit late-phase autophagy are different, and the structures of these three compounds are also much different. The elucidation of the functional mechanisms of spilacleoside as a late-phase autophagy inhibitor will be one of the most important issues in the future.

5. *"Similarly all of the experiments were performed on a single cell line, it*

*would be more informative if the author provided data from a second cell line"*

[Our response]

We mentioned in the “Introduction”, that the main aim of our study is to explore a means of regulating the expression levels of beneficial AMPs that does not directly apply inflammatory conditions or inflammatory factors to healthy human skin. Therefore, this study was performed using normal human epidermal keratinocytes (NHEKs) only. However, as you pointed out, we acknowledge the importance of examinations using a second cell line such as HaCaT cells to better understand RAE-induced RNase 7 expression. We would like to address your suggestion in our future research.

6. *Request for Image Clarity:*

*“I suggest improving the image quality of the colocalization experiments in Figure 3. In the current representation, it was challenging to discern the localization of LC3 and p62, which are pivotal for accurate interpretation.*

[Our response]

We have accepted your suggestion regarding the image quality of Fig 3D. Thus, to clarify the colocalization of LC3 and p62, we adjusted the resolution (quality) of the figure. Please check the revised Fig 3D (Fig 3E in the revised Fig 3). Translating TIFF images into PDF seems to degrade image quality. Therefore, the revised figure of the TIFF image (Fig 3E) is also displayed here (preferably, check the TIFF image itself).
