# Supplementary material for: Ruscus aculeatus extract promotes RNase 7 expression through ERK activation following inhibition of late-phase autophagy in primary human keratinocytes
Source: PLoS One. 2024 Dec 3;19(12):e0314873. doi: 10.1371/journal.pone.0314873 (PMC11614269; doi:10.1371/journal.pone.0314873)
Supplement: Attachment — Submitted filename: Response to Reviewers R2.docx [file pone.0314873.s004.docx]

**Response to Reviewers**

We wish to express our appreciation for the insightful comments that we received from the editor and reviewers. We feel that the comments have helped us to significantly improve our paper. Our responses to each of the review comments are highlighted in red both here and in our revised manuscripts.

**Additional Editor Comments:**

1) *My main point concerns the inhibition/activation of early autophagy and*

*effects of RAE on RNase7 expression. Indeed, reduced detection of RNase7 was observed after inhibition of early autophagy. I do not understand why a down-regulation of RNase7 should be expected after activation of early autophagy; I rather suspect an increased expression. Please clarify this point.*

[Our response]

It is well known that early-phase autophagy (basal autophagic activity) participates in the formation of autophagosomes. We first observed that the inhibition of SIRT1 receptor, which is involved in early-phase autophagy, by EX-527 (a SIRT1 receptor inhibitor), resulted in the inhibition of RAE-induced RNase 7 expression. Based on this observation, we concluded that early-phase autophagy is essential for RAE-induced RNase 7 expression. Conversely, our study demonstrated that RAE suppresses late-phase autophagy (namely, is an inhibitor of late-phase autophagy), resulting in the accumulation of autophagosomes due to the hindered progression of autophagy by RAE, as confirmed through flux assays and other experiments. These results suggest that expression of RNase 7 by RAE may require the accumulation of autophagosomes produced by early-phase autophagy (basal autophagic activity). If so, even in the presence of RAE, an inhibitor of late-phase autophagy, if early-phase autophagy is promoted beyond its original activity by resveratrol, an autophagy inducer, early-phase autophagy will exceed the inhibitory effect of RAE on late-phase autophagy. This implies that autophagy goes from an unprogressive state to a progressive state. Therefore, since autophagosomes are degraded and do not accumulate, RAE-induced RNase 7 expression in the presence of resveratrol is expected to be smaller than that of RAE alone. Indeed, when autophagy is accelerated by resveratrol, the expression of RNase 7 by RAE was suppressed. From the results of resveratrol, we concluded that the inhibition of late-phase autophagy (i.e., accumulation of autophagosomes) is essential for RAE-induced RNase 7 expression. Therefore, we believe that our experiment design using resveratrol, a SIRT1 receptor activator, is consistent. However, we have noticed that there are inadequate or inappropriate descriptions in the text related to the experiment using resveratrol. We apologize for this and have corrected the text as follows.

(page 17, lines 385–387 in our revised manuscript)

Taken together, we concluded that early-phase autophagy (basal autophagic activity), which participates in the formation of autophagosomes, is required for RNase 7 expression that is induced following RAE treatment.

(pages 17–18, lines 401–409 in our revised manuscript)

We observed that RAE inhibited late-phase autophagy and autophagosomes were accumulated by RAE treatment. However, it remains unclear whether the inhibition of late-phase autophagy by RAE (i.e., accumulation of autophagosomes) participated in RNase 7 expression. If it does, even in the presence of RAE, an inhibitor of late-phase autophagy, if early-phase autophagy is promoted beyond its original activity by an autophagy inducer, early-phase autophagy will exceed the inhibitory effect of RAE on late-phase autophagy. This implies that autophagy goes from an unprogressive state to a progressive state. Therefore, since autophagosomes are degraded and do not accumulate, RAE-induced RNase 7 expression in the presence of autophagy inducers is expected to be smaller than that of RAE alone.

For reference, the following is a schematic model to explain the mechanism of RAE-induced RNase 7 expression which we currently consider. We have already indicated this model in the "Response to Reviewers" of our first revised manuscript. Please forgive us if you have known this.

*Please explain the differences between letters a-d in Figures 2-6.*

[Our response]

Letters in Fig. 2-6 is one of methods that indicate statistical differences between the groups being compared. Different letters indicate significant differences between the groups being compared. On the other hand, same letters indicate no significant differences between the groups being compared. This explanation is described in each figure legend as follows.

(In each figure legend)

Different letters indicate significant differences based on post-hoc Tukey’s test results. P < 0.05.

2) *The entire blot corresponding to pp38 detection is of very poor quality*

*with many un-specific bands. Please provide a blot of better quality or delete the data related to p38 (Fig2D).*

[Our response]

In our study, since the expression levels of p-p38 was very small, many nonspecific bands probably appeared on the entire blot corresponding to p-p38 detection. This result strongly suggests that p-p38 is not involved in the expression of RNase 7 by RAE at all. Therefore, with reference to your suggestion, we deleted Fig 2B and have corrected our manuscript as follows.

(page 12, lines 269–271 in our revised manuscript)

RAE significantly increased the phosphorylation levels of ERK and JNK proteins compared with control (Figure 2A and 2B). Conversely, RAE did not affect the phosphorylation levels of p38 protein at all (data not shown).

3) *N=3; does this mean 3 technical replicates (same extract) or biological replicates (3 different cultures)? Please clarify.*

[Our response]

Thank you for your suggestion. The number (n) in figure legends of our manuscript indicates 3 biological replicates (3 different cultures). The description in figure legends was inappropriate, so it has been corrected as follows.

(In each figure legend)

Data represent the mean ± SD of three independent experiments.

**Reviewer #2**

[Our response]

Thank you very much for your time and effort taken to reviewing our manuscript.

**Journal Requirements:**

*Please review your reference list to ensure that it is complete and correct. If you have cited papers that have been retracted, please include the rationale for doing so in the manuscript text, or remove these references and replace them with relevant current references. Any changes to the reference list should be mentioned in the rebuttal letter that accompanies your revised manuscript. If you need to cite a retracted article, indicate the article’s retracted status in the References list and also include a citation and full reference for the retraction notice.*

[Our response]

We have confirmed your request. The retracted articles are not included in the references of our manuscript.
